# Palmitoyl acyltransferase DHHC21 mediates endothelial dysfunction in systemic inflammatory response syndrome

**DOI:** 10.1038/ncomms12823

**Published:** 2016-09-22

**Authors:** Richard S. Beard, Xiaoyuan Yang, Jamie E. Meegan, Jonathan W. Overstreet, Clement G.Y. Yang, John A. Elliott, Jason J. Reynolds, Byeong J. Cha, Christopher D. Pivetti, David A. Mitchell, Mack H. Wu, Robert J. Deschenes, Sarah Y. Yuan

**Affiliations:** 1 Department of Molecular Pharmacology and Physiology, Morsani College of Medicine, University of South Florida, Tampa, Florida 33612, USA; 2 Department of Surgery, Morsani College of Medicine, University of South Florida, Tampa, Florida 33612, USA; 3 Department of Surgery, School of Medicine, University of California at Davis, Sacramento, California 95817, USA; 4 Department of Molecular Medicine, Morsani College of Medicine, University of South Florida, Tampa, Florida 33612, USA; 5 James A. Haley Veterans' Hospital, Tampa, Florida 33612, USA

## Abstract

Endothelial dysfunction is a hallmark of systemic inflammatory response underlying multiple organ failure. Here we report a novel function of DHHC-containing palmitoyl acyltransferases (PATs) in mediating endothelial inflammation. Pharmacological inhibition of PATs attenuates barrier leakage and leucocyte adhesion induced by endothelial junction hyperpermeability and ICAM-1 expression during inflammation. Among 11 DHHCs detected in vascular endothelium, DHHC21 is required for barrier response. Mice with DHHC21 function deficiency (*Zdhhc21*^*dep/dep*^) exhibit marked resistance to injury, characterized by reduced plasma leakage, decreased leucocyte adhesion and ameliorated lung pathology, culminating in improved survival. Endothelial cells from *Zdhhc21*^*dep/dep*^ display blunted barrier dysfunction and leucocyte adhesion, whereas leucocytes from these mice did not show altered adhesiveness. Furthermore, inflammation enhances PLCβ1 palmitoylation and signalling activity, effects significantly reduced in *Zdhhc21*^*dep/dep*^ and rescued by DHHC21 overexpression. Likewise, overexpression of wild-type, not mutant, PLCβ1 augments barrier dysfunction. Altogether, these data suggest the involvement of DHHC21-mediated PLCβ1 palmitoylation in endothelial inflammation.

## Abstract (web summary)

Breaking down the endothelial barrier is a hallmark of systemic inflammatory response syndrome. Here the authors show that palmitoylation, a post-translational modification of proteins, plays a critical role in altering endothelial function during inflammation, and suggest the targeting of palmitoyl acyltransferase DHHC21 as potential disease therapy.

## Graphical abstract

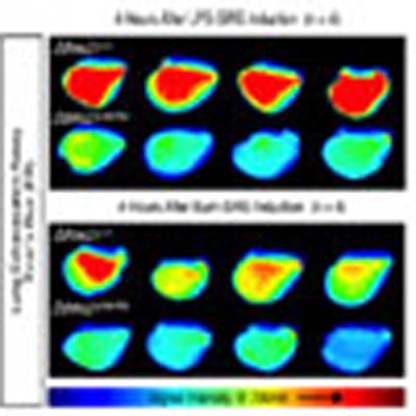

Many critically ill patients present with systemic inflammatory response syndrome (SIRS), one of the most challenging conditions resulting from infection (sepsis) or non-infectious injury (trauma, pancreatitis, haemorrhage)^1^,^2^,^3^. Despite improved patient care, their mortality and morbidity remain high owing to the development of multiple organ failure. Following injury, SIRS is triggered by an array of danger-associated molecular patterns and mediators released into the circulation, including histamine, thrombin and cytokines, most of which target vascular endothelium causing microvascular leakage, coagulopathy and leucocyte diapedesis^4^. Multiple etiologies and complex receptor-signalling pathways are attributed to this disease state; however, therapies directed at targeting the upstream of inflammatory cascades (cytokine inhibitors, histamine antagonists, so on) show limited efficacy^1^,^3^,^5^. Likewise, blockades of innate immunity (TLR4 antagonist) or coagulation (anti-thrombin) fail to demonstrate improved survival^6^,^7^. Recently, recognizing the central role of endothelial dysfunction in inflammation, the FDA approved recombinant human activated protein C for treating sepsis. Unfortunately, it was quickly withdrawn due to minimal mortality benefit and increased bleeding risk^8^. Currently, several new therapies, including anti-HMGB1 and sphingosine-1-phosphate analogous are being investigated^1^,^3^,^9^,^10^; many of them are not designed to specifically treat endothelial dysfunction. The underdevelopment of endothelial-targeting interventions highlights the need for further studies.

A hallmark of endothelial dysfunction during SIRS is opening of cell–cell junctions promoting plasma and leucocyte extravasation. Because barrier breakdown or loosening often occurs rapidly (within minutes) after stimulation, it is plausible that dynamic modifications of the endothelial barrier serve as the major mechanism for vascular hyperpermeability^11^,^12^,^13^. Many studies have focused on phosphorylation of molecules involved in the initial establishment of barrier structure or upstream signalling such as kinase receptors^14^,^15^,^16^. However, it is becoming increasingly apparent that phosphorylation alone may not account for all signalling or structural changes in the barrier under infectious or injurious conditions^17^,^18^,^19^. The purpose of this study is to evaluate our hypothesis that another dynamic regulatory mechanism, namely protein palmitoylation, plays a critical role in altering endothelial function during inflammation.

Palmitoyl acyltransferases (PATs) are a family of enzymes that catalyse protein S-palmitoylation, a thioester linkage of palmitic acid to cysteine residues. All PATs share a signature catalytic domain containing a zinc finger aspartate-histidine-histidine-cysteine (DHHC) motif^20^. Since the initial discovery of a DHHC-PAT in yeast by Roth^21^ and Deschenes^22^, the DHHC family has grown to 23 members in human and 24 in mouse genomes. Functionally, PAT-catalysed palmitoylation is shown to rapidly change protein locations, activity and ability to interact with other proteins^23^. Recent studies of palmitoylation kinetics have underscored the potential of PATs as a ‘kinase-like' factor in signal transduction^24^,^25^,^26^,^27^. So far, the biologic effects of PATs have been primarily investigated in the fields of cell growth, lipid metabolism and neuroscience. Studies have also reported their involvement in Wnt-driven carcinogenesis^28^, insulin-promoted angiogenesis^29^, neurologic diseases^30^ and metabolic disorders^31^,^32^. Most recently, an *in vivo* study demonstrated a role for DHHC21-PAT in mediating vasoconstriction by palmitoylating α-adrenergic receptor^33^, suggesting the possibility that PATs serve as an important dynamic regulator of circulatory functions.

Since there is currently no direct evidence for PATs regulating endothelial function during inflammation, we sought to determine if and how PATs affect vascular barrier properties and leucocyte-endothelium interactions under SIRS relevant conditions, using animal models of systemic inflammation caused by traumatic injury (burns) and septic insult (LPS injection), as well as microvessels and endothelial monolayers stimulated by typical inflammatory mediators (histamine, thrombin or IL-1β). Four sets of experiments were designed to study the DHHC21-PLCβ1 pathway in endothelial inflammation. In the first set of experiments, we evaluated the effects of general pharmacologic inhibition of PATs *in vivo* during trauma or endotoxemia and *in vitro* during inflammatory stimulation. We also verified the underlying cellular responses focusing on endothelial cell–cell junction permeability and ICAM-1 expression. In the second set of experiments, after confirming the expression of individual PATs in vascular endothelium, we conducted a functional screening in endothelial monolayers subjected to individual gene knockdown to determine that the presence of specific PATs, namely DHHC21, is required for barrier responses. In the third set of experiments, we used a genetically modified mouse model of DHHC21 function deficiency (*Zdhhc21*^*dep/dep*^) to evaluate the contribution of this PAT to SIRS-induced organ injury and mortality. The relative importance of endothelial-specific DHHC21 was also evaluated. In the fourth set of experiments, we identified PLCβ1 as a target of DHHC21. Further mechanistic studies were directed at measuring PLCβ1 palmitoylation, subcellular distribution and signalling activity in *Zdhhc21*^*dep/dep*^ cells. Moreover, comparisons were made in endothelial cells subjected to PLCβ1 knockdown, PLCβ1 wild-type overexpression and PLCβ1 C17 mutant overexpression to determine the mechanistic contribution of PLCβ1 to endothelial dysfunction. The results from these experiments show that protein palmitoylation plays a critical role in mediating aberrant activation of vascular endothelium during inflammation. We suggest targeting DHHC21 as potential therapy of inflammatory diseases.

## Results

### PAT inhibition prevents vascular barrier breakdown

**Figure 1:**
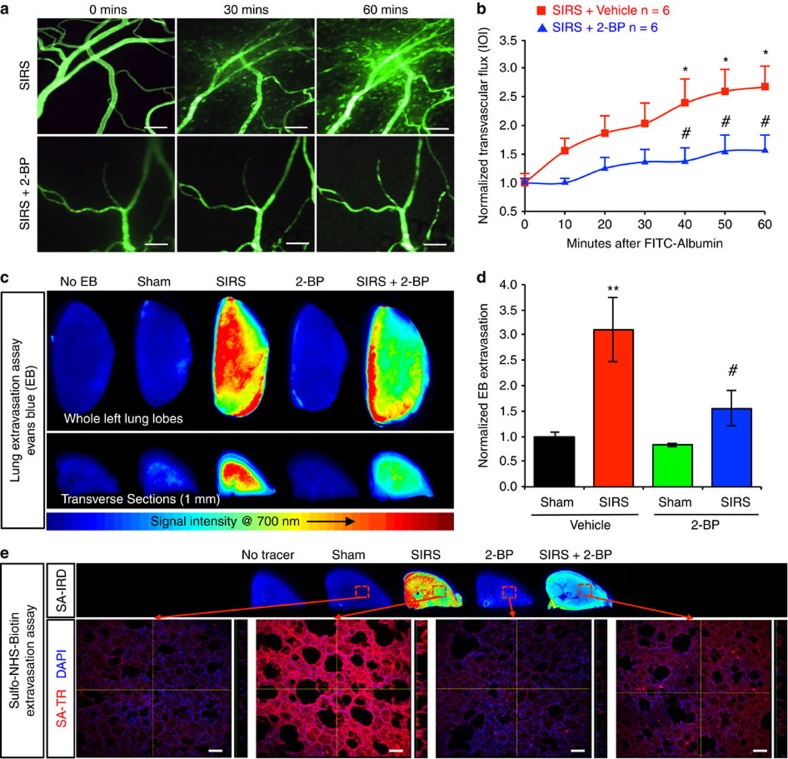
Inhibition of PATs attenuates microvascular leakage during systemic inflammatory response to burn injury. The palmitoyl acyltransferase inhibitor 2-bromopalmitate (2-BP, 2 mg kg^−1^) was intravenously administrated 20 min prior to SIRS induction. (**a**,**b**) Transvascular flux of FITC-albumin from mesenteric microvessels observed via intravital microscopy 3 h postburn. (**a**) Representative images (Scale bars, 100 μm). (**b**) Quantification of transvascular flux (*n*=number of animals). For each group, values were normalized to its own *t*=0 min and presented as mean±s.e.m. *P<0.05 versus sham+vehicle, ^#^*P*<0.05 versus SIRS+vehicle. (**c**,**d**) Plasma protein leakage in the lungs measured as tracer Evans Blue extravasation. (**c**) Representative images showing tracer leakage captured at 700 nm with infrared imaging scanner and pseudo-colored with a heat-map colour scheme. (**d**) Quantitative extravasation assay (*n*=6). Values were normalized to vehicle-treated group subjected to sham operation and presented as mean±s.e.m. **P<0.01 versus sham+vehicle, ^#^*P*<0.05 versus SIRS+vehicle. (**e**) Microvascular leakage in the lungs measured as interstitial deposition of small molecule tracer sulfo-NHS-biotin. Upper panels: representative transverse lung slices (500 μm) probed for extravasated sulfo-NHS-biotin with IRDye 800-conjugated streptavidin (SA-IRD) and scanned with infrared imaging system (800 nm). Images were pseudo-colored with heat-map colour scheme (blue=low intensity, red=high intensity). Lower panels: representative confocal micrographs of lung sections probed for sulfo-NHS-biotin with Texas Red-streptavidin (SA-TR). Nuclei were stained with DAPI. Scale bars, 100 μm.

Two-bromopalmitate (2-BP) is a general inhibitor of PAT activity that acts by suppressing the formation of acyl-intermediates^34^. To determine the pathophysiological involvement of PATs in inflammation, we tested the effects of 2-BP to reduce microvascular leakage. In healthy rats, intravenous administration of 2-BP did not cause mortality or obvious abnormalities in cardiorespiratory function or mesenteric circulation for the duration of our experiments. However, animals receiving 2-BP prior to SIRS induction displayed drastically reduced plasma extravasation through mesenteric vessels indicated by transvascular flux of FITC-albumin (Fig. 1a,b). Moreover, 2-BP ameliorated vascular barrier injury in the lungs, measured by tissue uptake of Evans Blue, a marker of albumin leakage (Fig. 1c,d), and by interstitial deposition of sulfo-NHS-biotin, a marker of small molecule efflux (Fig. 1e).

**Figure 2:**
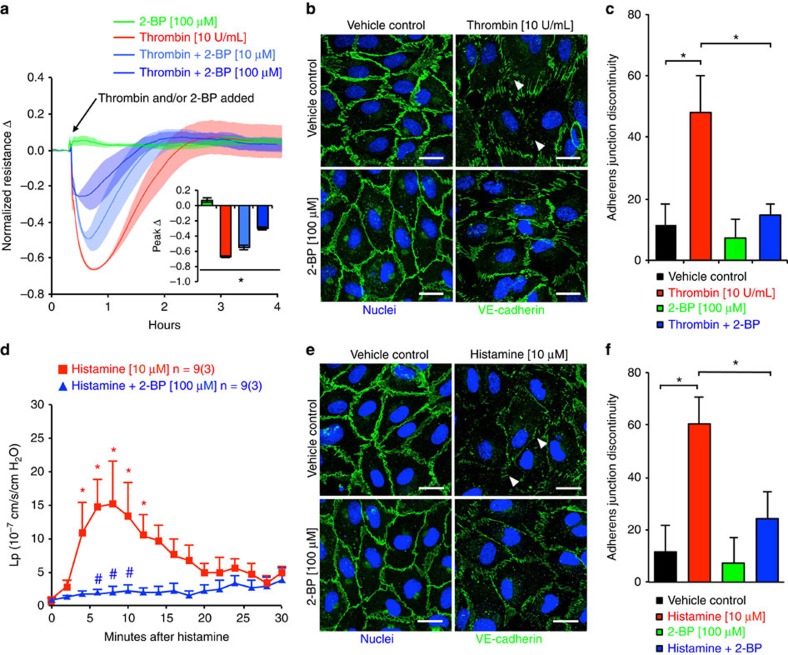
Inhibition of PATs reduces inflammatory mediator-induced endothelial cell–cell junction hyperpermeability. (**a**) Time- and dose-dependent effects of 2-BP on thrombin (10 U ml^−1^)-induced reduction in transendothelial electric resistance (TER), an indicator of cell–cell adhesive barrier function. Values at each time point were normalized to their baseline at *t*=0. Solid line tracing represents the mean resistance, and shadow represents standard error. Embedded graph indicates peak TER values and presented as mean±s.e.m. (*n*=3 independent experiments). *P<0.05. (**b**) Representative confocal images of the adherens junction molecule VE-cadherin (green) and nuclei (blue) 30 min after thrombin with or without pretreatment of 2-BP. Arrows point to discontinued VE-cadherin strands or intercellular gaps. Scale bars, 10 μm. (**c**) Quantification analysis of VE-cadherin junction discontinuity from three independent experiments, presented as mean±s.e.m. *P<0.05. (**d**) Histamine-induced changes in hydraulic conductivity (*L*_p_) in intact perfused venules without or with 2-BP (given 30 min prior to histamine and continuously perfused until the end of the observation). *L*_p_ indicates venular permeability to water which transports mainly via paracellular pathway. *n*=number of segment measurements; values in parentheses indicate number of independent experiments. *P<0.05 versus baseline, ^#^*P*<0.05 versus histamine+vehicle. (**e**) Representative confocal images of VE-cadherin (green) and nuclei (blue) 10 min after histamine in endothelial cells pre-treated with 2-BP. Arrows point to discontinued VE-cadherin strands or intercellular gaps. Scale bars=10 μm. (**f**) Quantification analysis of VE-cadherin junction discontinuity from three independent experiments. Data are presented as mean±s.e.m. *P<0.05.

We then examined the effects of PAT inhibition on endothelial cell–cell adhesive barrier morphology and function in human umbilical vein endothelial cells (HUVEC) during inflammatory stimulation. Consistent with our *in vivo* findings, 2-BP inhibited barrier dysfunction, indicated by reduced transendothelial electrical resistance (indicator of cell–cell adhesive barrier property) in response to thrombin in a time-dependent and dose-related manner (Fig. 2a). Given that the barrier integrity of microvascular endothelium is largely determined by adherens junctions (AJs)^35^, we focused on the changes in VE-cadherin, the primary AJ molecule. As shown in Fig. 2b, VE-cadherin staining under unstimulated conditions was characterized by continuous distribution at endothelial cell–cell contacts. Upon inflammatory stimulation by thrombin (Fig. 2b), it became discontinuous, diffuse or thinner. Intercellular gaps were formed (arrows). Quantitative results showed that 2-BP significantly reduced thrombin-induced junction discontinuity (Fig. 2c). Similar inhibitory effects of 2-BP on histamine-induced microvascular hyperpermeability (Fig. 2d) and junction dissociation were observed (Fig. 2e,f). Further, we confirmed the direct effects of 2-BP on intact postcapillary venules, which are known to serve as the major vascular bed for plasma leakage and leucocyte diapedesis during inflammation. Using a modified Landis micro-occlusion technique, we measured hydraulic conductivity (*L*_p_; indicator of permeability to water filtration along pressure gradient) in single perfused venules. Perfusion of 2-BP significantly reduced the increase in *L*_p_ caused by histamine (Fig. 2d), consistent with 2-BP attenuation of histamine-induced AJ disruption *in vitro* (Fig. 2e,f).

### PAT inhibition reduces leucocyte adhesion/ICAM-1 expression

**Figure 3:**
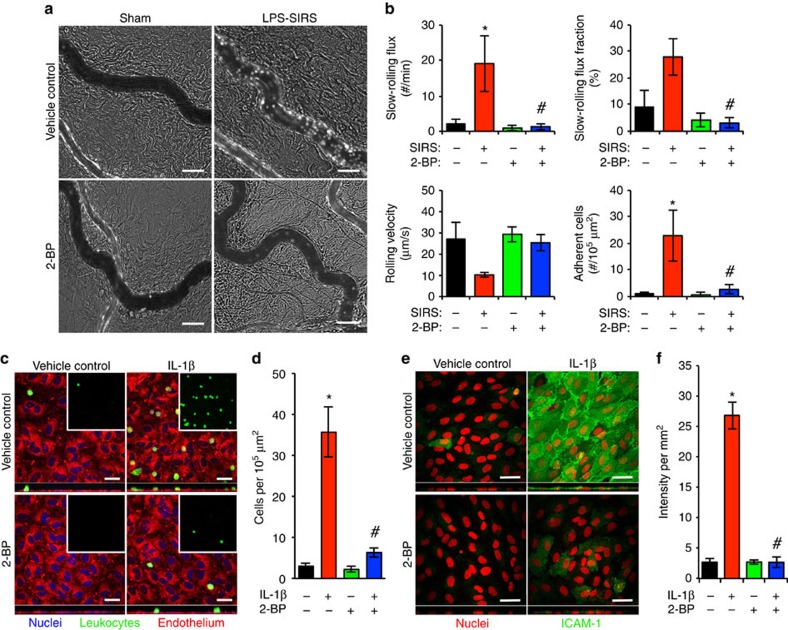
PAT inhibition suppresses leucocyte adhesion and ICAM-1 expression on endothelial surface during inflammatory stimulation. (**a**,**b**) Leucocyte-endothelium interactions were assessed via intravital microscopic analysis of mesenteric microcirculation in rats subjected to SIRS elicited by bacterial lipopolysaccharide (LPS, 10 mg kg^−1^, IP, 4 h). A group of animals received intravenous 2-BP (2 mg kg^−1^) immediately after LPS injection. Leucocytes were labelled with acridine orange. (**a**) Representative images of leucocyte adhesion in postcapillary venules. Scale bars, 30 μm. (**b**) Quantification of leucocyte slow-rolling flux (number of cells per min), slow-rolling fraction (% of slow rolling cells to total rolling cells), rolling velocity and adhesion. Increased slow-rolling flux or decreased rolling velocity indicates the likelihood of firm adhesion. Data are presented as mean±s.e.m. from 4 rats with ≥10 venules per group. *P<0.05 versus sham+vehicle, ^#^*P*<0.05 versus SIRS+vehicle. (**c**,**d**) The effects of 2-BP on human leucocyte adhesion to human umbilical vein endothelial cells with or without stimulation by IL-1β (100 ng ml^−1^, 4 h). 2-BP (10 μM) or vehicle was given simultaneously with IL-1β. (**c**) Representative images of adherent leucocytes (green) on endothelial cells (red) with nuclei stained with DAPI. Embedded images show green channel (leucocytes) alone. Scale bars, 25 μm. (**d**) Quantification of adherent leucocytes to IL-1β-stimulated ECs. Bar graph represents mean±s.e.m. from three independent experiments. *P<0.05 versus unstimulated+vehicle, ^#^*P*<0.05 versus IL-1β+vehicle. (**e**,**f**) The effects of 2-BP on IL-1β-induced ICAM-1 expression on endothelial surface. Endothelial cells were treated with 100 ng ml^−1^ IL-1β for 4 h with or without 10 μM 2-BP (given simultaneously with IL-1β). (**e**) Representative confocal images. Scale bars, 50 μm. (**f**) Quantification of ICAM-1 surface expression. Results represent mean±s.e.m. from three independent experiments. *P<0.05 versus unstimulated+vehicle, ^#^*P*<0.05 versus IL-1β+vehicle.

Leucocyte-endothelial interactions were observed in rat mesenteric microvessels via intravital microscopy (Fig. 3a). During inflammation slow-rolling leucocytes are more likely to be arrested by endothelial cells leading to firm adhesion. We thus evaluated the effects of PAT inhibition on leucocyte adhesion along with their slow-rolling flux, slow-rolling fraction and rolling velocity. As shown in Fig. 3b, SIRS significantly increased leucocyte adhesion and slow rolling. Compared with vehicle-treated animals, 2-BP-pretreated animals demonstrated an attenuated response in leucocyte adhesion. Additionally, adhesion of human leucocytes to HUVEC monolayers after stimulation by interleukin-1β (IL-1β) was measured. In basal conditions or cells treated with 2-BP alone, minimal adhesion was observed. However, after IL-1β stimulation, there was a significant increase in the number of adherent leucocytes; the response was attenuated by 2-BP treatment (Fig. 3c,d). Because ICAM-1 expression on the surface of endothelium is required for leucocyte adhesion, we also tested the efficacy of 2-BP to reduce ICAM-1 surface expression under these conditions. Indeed, there was a significant increase in endothelial cell surface expression of ICAM-1 in response to IL-1β, a response prevented by 2-BP (Fig. 3e,f). Altogether, these findings indicate the involvement of DHHC-PATs in vascular inflammation.

### Inflammation increases PLCβ1 palmitoylation and signalling

**Figure 4:**
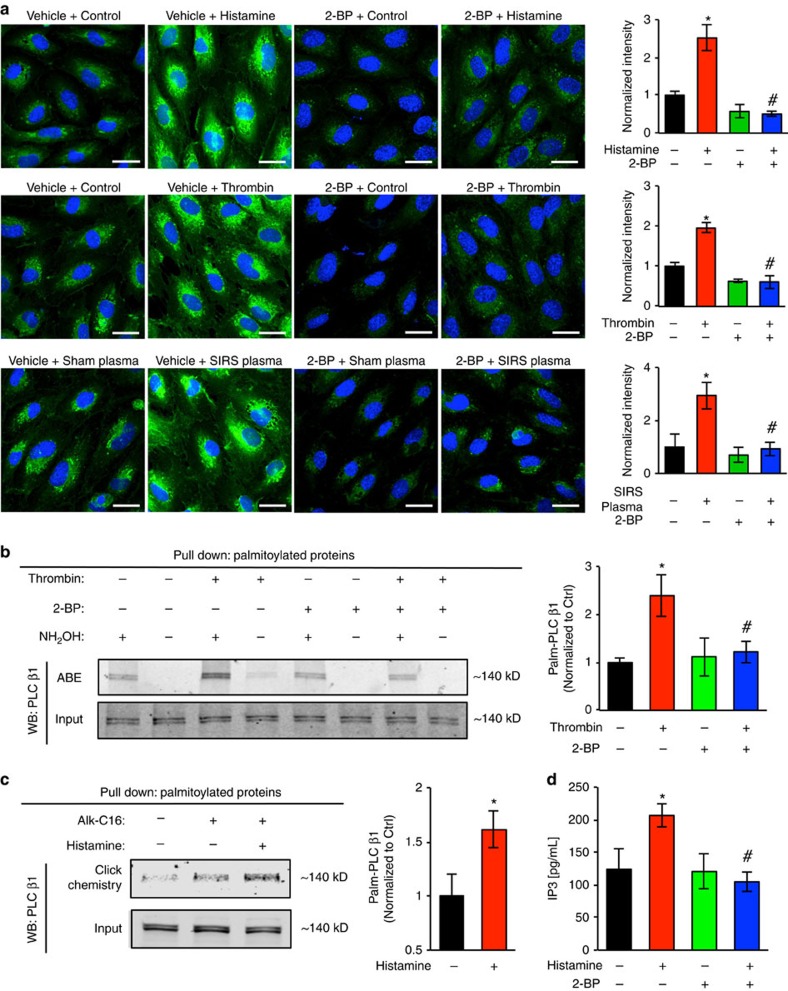
Inflammatory stimuli increase PLCβ1 palmitoylation and its signaling activity. (**a**) Increased palmitoylation was detected in endothelial cells after stimulation with SIRS plasma or inflammatory stimuli. Representative images of palmitoylation in cells treated with vehicle, histamine (10 μM, 15 min), thrombin (10 U ml^−1^, 30 min) or SIRS plasma (20% dilution, 1 h) in the absence or presence of 2-BP (pretreatment at 10 μM). Palmitoylated proteins were metabolically labelled with a palmitic acid analogue ω-alkynyl palmitic acid (Alk-C16). Labelled proteins were visualized by further probing with fluorescent azide via Click chemistry reaction. Palmitoylated proteins are shown in green and nuclei blue. Scale bar, 30 μm. Bar graph shows quantification of total palmitoylation signal intensity. Results are from three independent experiments presented as mean±s.e.m. *P<0.05 versus control+vehicle, ^#^*P*<0.05 versus stimulus+vehicle. (**b**,**c**) The levels of palmitoylated PLCβ1 in endothelial cells were determined using two different methods. (**b**) Acyl-biotin exchange. Whole cell lysates from thrombin (10 U ml^−1^) or vehicle treated ECs, with or without 2-BP, were subject to acyl-biotin exchange to isolate palmitoylated proteins in the presence of hydroxylamine (NH_2_OH). The samples were then used in Western blotting for PLCβ1. Band intensity was quantified and normalized to control. Results represent three independent experiments. *P<0.05 versus vehicle+control, ^#^*P*<0.05 versus vehicle+thrombin. (**c**) Pull-down of palmitoylated proteins and immunoblotting for PLCβ1. ECs were incubated with Alk-C16 overnight and then treated with histamine (10 μM) or vehicle. Whole cell lysates were collected, and palmitoylated proteins were pulled down using Azide Agarose Resin via Click chemistry reaction. Palmitoylated protein samples were subject to Western blotting for PLCβ1. Band intensity was quantified and normalized to control group. Bar graph represents four independent experiments. *P<0.05 versus vehicle. (**d**) IP3 levels in ECs subjected to histamine stimulation (10 μM) with or without 2-BP (10 μM). Bar graph represents mean±s.e.m. from three independent experiments. *P<0.05 versus vehicle, ^#^*P*<0.05 versus vehicle+histamine.

In an effort to quantify protein palmitoylation in endothelial cells, we employed Click chemistry to measure the level of total palmitoylated proteins in HUVECs under basal conditions and during stimulation by SIRS plasma or inflammatory agents (thrombin, histamine). Palmitoylated proteins were metabolically labelled with a palmitic acid analogue, ω-alkynyl palmitic acid (Alk-C16), and further probed with fluorescent Oregon Green 488 azide by copper-catalysed click reaction for observation under confocal microscopy. The data showed that following inflammatory stimulation by histamine, thrombin or SIRS plasma, there was a significant increase in total palmitoylated proteins. Treatment with 2-BP prevented this response (Fig. 4a).

We then tried to identify specific proteins that undergo palmitoylation in inflammation. In initial screening, we conducted a proteomic analysis combining acyl-biotin exchange (ABE) with LC–MS/MS. ABE is a series of biochemical reactions involving the exchange of protein palmitoylation moieties with biotin, which can be detected via pull-down with streptavidin beads. We identified a protein that undergoes increased palmitoylation in endothelial cells upon SIRS stimulation, phospholipase C β1 (PLCβ1), an important signalling molecule downstream of the G-protein coupled receptors (GPCR) known to mediate the effects of inflammatory mediators such as thrombin and histamine. Thus, we sought to focus on PLCβ1 in the following experiments. Our data demonstrated that thrombin induced a significant increase in PLCβ1 palmitoylation, which was inhibited by 2-BP treatment (Fig. 4b) (see full-scanned western blots and gels in Supplementary Figs 1–5). Consistently, a pull-down assay with azide agarose resin based on metabolic labelling and Click chemistry showed that histamine stimulation was able to increase the level of PLCβ1 palmitoylation (Fig. 4c). Since it is well established that PLCβ1 activation leads to IP3 production, we next examined the concomitant changes in IP3 levels as an indicator of PLCβ1 signalling activity. The results showed that histamine increased IP3 production and this effect was not observed in cells treated with 2-BP (Fig. 4d), suggesting the potential involvement of palmitoylation-coupled PLCβ1 signalling activity in inflammation.

### DHHC5/21 are critical in endothelial barrier response

**Figure 5:**
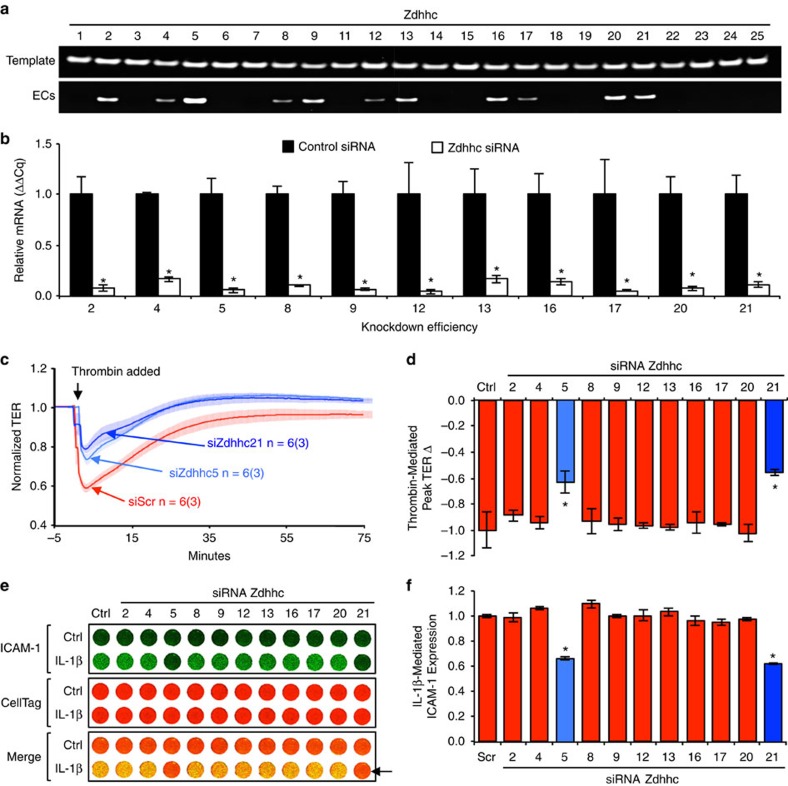
Endothelial-expressing *Zdhhcs* and importance of DHHC5/21 in endothelial dysfunction. (**a**) Representative images from qualitative PCR analyses of 24 known murine *Zdhhcs* in primary MLMVECs that confirmed the expression of 11 *Zdhhcs*. cDNA template of each gene was used as a positive control. (**b**) Knockdown efficiency of the 11 *Zdhhcs* expressed in endothelial cells verified by RT-PCR, *P<0.05 versus control siRNA. (**c**,**d**) The effects of individual *Zdhhc* knockdown on thrombin-induced endothelial barrier dysfunction indicated by TER. (**c**) Representative TER tracings of *Zdhhc5* and *Zdhhc21* knockdown in response to thrombin (10 U ml^−1^). Solid line tracing represents the mean resistance, and shadow represents±s.e.m. *n*=number of measurement; value in parentheses indicates number of independent experiments. (**d**) Quantification of peak TER reduction upon thrombin treatment in each *Zdhhc* knockdown group. Values were normalized to TER with control siRNA. Bar graph represents mean±s.e.m. from three independent experiments. *P<0.05 versus control siRNA. (**e**,**f**) The effects of individual *Zdhhc* knockdown on IL-1β-induced ICAM-1 expression on EC surface. MLMVECs in a 96-well plate were stimulated with IL-1β (100 ng ml^−1^, 4 h). ICAM-1 surface expression was determined by on-cell western assay using Odyssey CLx Infrared Imaging System. (**e**) Representative images. CellTag staining was used for cell number normalization. Arrow indicates decreased ICAM-1 expression. (**f**) Quantification of ICAM-1 expression in *Zdhhc* knockdown ECs upon IL-1β stimulation. Results represent mean±s.e.m. from three independent experiments. *P<0.05 versus control siRNA.

More than 20 DHHC-PATs have been identified (Supplementary Table 1) and are known to have heterogeneous tissue distribution^36^; however, their expression profile in the vascular endothelium has not been fully reported. In this study, we evaluated DHHC-PAT expression and function in murine lung microvascular endothelial cells (MLMVECs). Quantitative PCR was performed to characterize endothelial-specific *Zdhhc* gene expression (Fig. 5a). Among the 24 known *Zdhhcs*, only *Zdhhcs 2, 4, 5, 8, 9, 12, 13, 16, 17, 20* and *21* were detected. To investigate which of these may regulate endothelial injury, we silenced their expression individually with siRNA and assessed the functional impact on thrombin-mediated endothelial barrier dysfunction and IL-1β upregulation of ICAM-1 expression. The efficiency of the siRNA knockdown was confirmed by RT-PCR (Fig. 5b). Out of the 11 endothelial-expressing *Zdhhcs*, only knockdown of *Zdhhc5* or *Zdhhc21* significantly ameliorated barrier dysfunction (Fig. 5c,d) and ICAM-1 expression (Fig. 5e,f). These data demonstrate an important role for DHHC5- and DHHC21-PATs in endothelial response to inflammatory stimulation.

### *Zdhhc21^dep/dep^* mice are resistant to organ injury in SIRS

**Figure 6:**
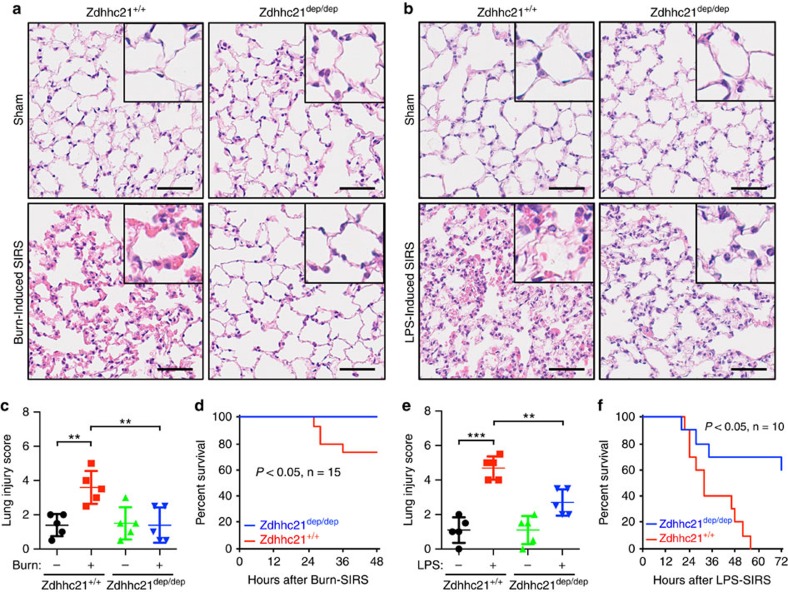
*Zdhhc21*^*dep/dep*^ mice are resistant to SIRS-induced lung injury and mortality. (**a**,**b**) Representative images of lung tissues obtained from mice 24 h after thermal injury-induced (**a**) or LPS-induced SIRS (**b**) (five mice in each group). Scale bar, 50 μm. (**c**) Lung histology injury scores in thermal injury-induced SIRS. Results represent mean±s.e.m. from five mice. **P<0.01. (**d**) Survival rates in mice subjected to thermal injury (*n*=15 mice). (**e**) Lung histology injury scores in LPS-induced SIRS. Results represent mean±s.e.m. from five mice. **P<0.01, ***P<0.001. (**f**) Survival rates in mice subjected to LPS challenge (*n*=10 mice).

After determining the importance of DHHC21 in inflammation-mediated endothelial injury, we focused on *in vivo* studies utilizing a DHHC21 deficient mouse model, *Zdhhc21*^dep/dep^. Its genetic background involves a spontaneous 3bp deletion in the coding region of the *Zdhhc21* gene rendering DHHC21 function deficiency^37^. The effect of DHHC21 deficiency on overall SIRS outcomes was determined. Specifically, lung injury 24 h after SIRS-induction was evaluated by histological H&E staining in both *wild-type* controls (*Zdhhc21*^*+/+*^) and *Zdhhc21*^*dep/dep*^ mice. In *Zdhhc21*^*+/+*^ mice, thickening of alveoli-capillary membrane and leucocyte infiltration were observed; these pathologies were significantly ameliorated in *Zdhhc21*^*dep/dep*^ mice (Fig. 6a–c,e). Furthermore, as shown in Fig. 6d, thermal injury-induced SIRS caused ∼30% mortality in *Zdhhc21*^*+/+*^ mice by 48 h, whereas no deaths were observed in *Zdhhc21*^*dep/dep*^ mice during the same time course. Similarly, as shown in Fig. 6f, LPS-induced SIRS caused 100% mortality of *Zdhhc21*^*+/+*^ mice within 60 h, whereas there was still a 60% survival rate in *Zdhhc21*^*dep/dep*^ mice at 72 h.

### Endothelial DHHC21 is important for leucocyte adhesion

**Figure 7:**
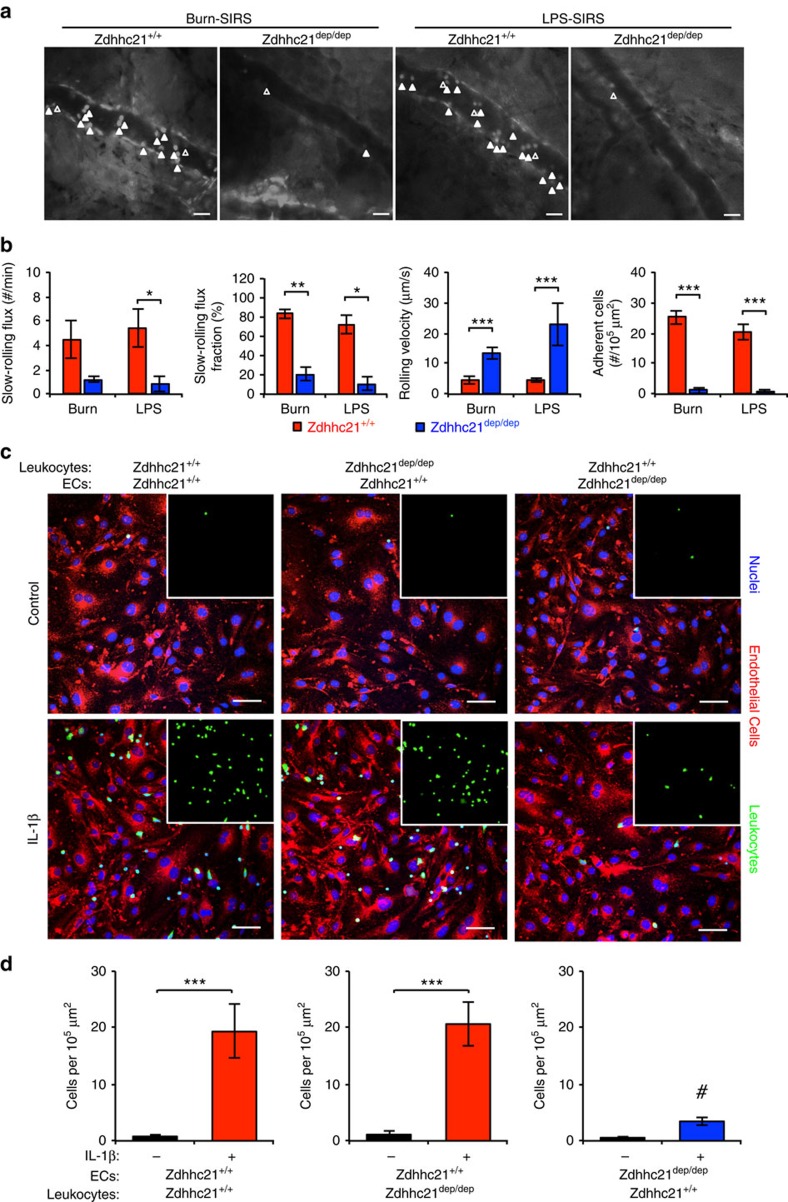
DHHC21 deficiency attenuates leucocyte-endothelial adhesion during inflammation. (**a**) Representative images of intravital microscopic analyses of microvessels in mouse ears during burn or LPS challenge. Scale bars, 50 μm. Solid arrowheads point to adherent leucocytes; open arrowheads point to slow-rolling leucocytes. (**b**) Quantification of leucocyte slow-rolling flux, slow-rolling fraction, rolling velocity and adhesion in *Zdhhc21*^*+/*+^and *Zdhhc21*^*dep/dep*^ mice 4 h postburn or LPS. Results represent mean±s.e.m. from six mice. *P<0.05, **P<0.01, ***P<0.001. (**c**,**d**) Comparative analysis of the leucocyte adhesion in endothelial versus leucocytic *Zdhhc21*^*dep/dep*^ using a chimeric approach. Leucocytes were isolated from either *Zdhhc21*^*+/+*^ or *Zdhhc21*^*dep/dep*^ and then applied to endothelial cells from either *Zdhhc21*^*+/+*^ or *Zdhhc21*^*dep/dep*^ and stimulated with IL-1β (100 ng ml^−1^, 4 h). (**c**) Representative confocal images of adherent leucocytes. Embedded images show leucocyte channel alone. Nuclei were stained with DAPI. Scale bars, 100 μm. (**d**) Quantification of adherent leucocytes shows that endothelial cells from *Zdhhc21*^*dep/dep*^ displayed marked resistance to leukocyte adhesion, whereas leucocytes from these mice did not show altered adhesiveness. Results present mean±s.e.m. from three independent experiments. ***P<0.001 versus unstimulated. ^#^*P*<0.05 versus IL-1β+*Zdhhc21*^*+/+*^ EC+*Zdhhc21*^*+/+*^ leukocyte.

To evaluate the effects of DHHC21 deficiency in leucocyte-endothelial interactions, we measured leucocyte rolling and adhesion in postcapillary venules of *Zdhhc21*^*+/+*^ and *Zdhhc21*^*dep/dep*^ mouse ears. *Zdhhc21*^*dep/dep*^ mice displayed basal circulatory characteristics comparable to *wild-type* controls, but with a blunted vascular injurious response to SIRS. In *Zdhhc21*^*+/+*^ mice subjected to burn- or LPS-induced SIRS, the number of slow-rolling leucocytes, the fraction of slow-rolling leucocytes and leucocyte adhesion to the microvessel wall were significantly increased, and the average leucocyte rolling velocity was greatly reduced; however, *Zdhhc21*^*dep/dep*^ mice were resistant to these responses (Fig. 7a,b).

Furthermore, primary MLMVECs and leucocytes were isolated from *Zdhhc21*^*+/+*^ and *Zdhhc21*^*dep/dep*^ mice to characterize the cell-specific effects of DHHC21 deficiency contributing to the reduced leucocyte-endothelial interactions. *Wild-type* leucocytes adhered normally to IL-1β activated *wild-type* ECs, but adhesion to *Zdhhc21*^*dep/dep*^ ECs was greatly attenuated. Interestingly, *Zdhhc21*^*dep/dep*^ leucocyte adherence to *wild-type* ECs was unaffected, indicating the relative importance of endothelial DHHC21 in this process (Fig. 7c,d).

### DHHC21 deficiency ameliorates endothelial barrier dysfunction

**Figure 8:**
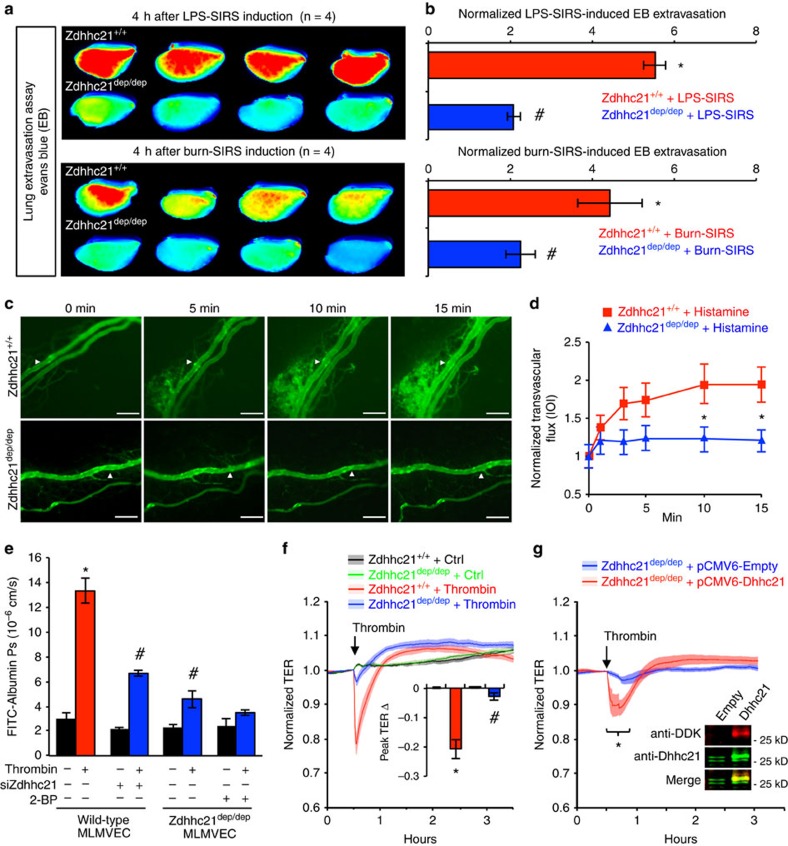
DHHC21 deficiency prevents inflammation-mediated vascular barrier dysfunction. (**a**,**b**) Following LPS or thermal injury, *Zdhhc21*^*dep/dep*^ mice displayed decreased plasma protein extravasation in the lungs. (**a**) Images of four replicates used to qualitatively demonstrate SIRS-induced Evans Blue extravasation in whole left lung lobes (signal intensity low=blue, high=red). (**b**) Quantitative results of Evans Blue extravasation assays. Values were normalized to *Zdhhc21*^*+/+*^mice receiving sham operation. Results represent mean±s.e.m. from four independent experiments. *P<0.05 versus *Zdhhc21*^*+/+*^+sham, ^#^*P*<0.05 versus *Zdhhc21*^*+/+*^+SIRS. (**c**,**d**) Histamine-induced transvascular flux of FITC-albumin from mesenteric microvessels. (**c**) Representative images at 0, 5, 10 and 15 min after histamine application. Scale bars, 100 μm. Arrowheads indicate the observed microvessel. (**d**) Quantification of plasma transvascular flux. For each genotype, value was normalized to its own *t*=0 min. Results represent mean±s.e.m. from six mice. *P<0.05 versus *Zdhhc21*^*+/+*^+histamine. (**e**) The effects of *Zdhhc21* knockdown or loss-of-function on endothelial permeability to FITC-albumin with or without 2-BP (100 μM). Thrombin (10 U ml^−1^) was applied to MLMVEC monolayers and their permeability was calculated based on albumin transendothelial diffusion rate. Bar graph represents mean±s.e.m. at *n*≥3. *P<0.05 versus *Zdhhc21*^*+/+*^ EC+vehicle, ^#^*P*<0.05 versus *Zdhhc21*^*+/+*^ EC+thrombin. (**f**) Dynamic changes of endothelial barrier resistance to thrombin (10 U ml^−1^) in MLMVECs isolated from *Zdhhc21*^*+/+*^ and *Zdhhc21*^*dep/dep*^ mice. TER value of each time point is normalized to baseline. Solid line tracing represents the mean resistance, and shadow represents standard error. Embedded graph shows maximum TER changes. Bar graph represents mean±s.e.m. *P<0.05 versus *Zdhhc21*^*+/+*^ EC+vehicle , ^#^*P*<0.05 versus *Zdhhc21*^*+/+*^ EC+thrombin. (**g**) Blunted TER response to thrombin (10 U ml^−1^) was partially rescued in cells overexpressing wild-type DHHC21. *Zdhhc21* (DDK tagged) or empty vector was overexpressed in *Zdhhc21*^*dep/dep*^ ECs. Results represent mean resistance±s.e.m. The efficiency of *Zdhhc21* overexpression was verified by Western blotting using anti-DDK tag and anti-DHHC21 antibody (Embedded graph).

The vascular barrier function was also determined in *Zdhhc21*^*dep/dep*^ mice during inflammation. Upon LPS or burn-induced SIRS, less protein leakage from lung microvessels indicated by interstitial Evans Blue intensity was detected in *Zdhhc21*^*dep/dep*^ mice compared with that in *Zdhhc21*^*+/+*^ mice (Fig. 8a,b). Consistently, following histamine stimulation, increased FITC-albumin leakage was observed in mesenteric vessels of *Zdhhc21*^*+/+*^ mice, but this response was significantly reduced in vessels of *Zdhhc21*^*dep/dep*^ mice (Fig. 8c,d).

The key role of endothelial DHHC21 in regulating barrier dysfunction was further confirmed in our *in vitro* inflammatory models. As shown in Fig. 8e, *Zdhhc21* knockdown MLMVECs and *Zdhhc21*^*dep/dep*^ ECs exhibited attenuated hyperpermeability to FITC-albumin upon thrombin stimulation, and the endothelial hyperpermeability was nearly completely blocked in *Zdhhc21*^*dep/dep*^ ECs treated with 2-BP. *Zdhhc21*^*dep/dep*^ ECs also displayed significantly reduced transendothelial electric resistance (TER) response to thrombin (Fig. 8f); however overexpression of *wild-type Zdhhc21* in *Zdhhc21*^*dep/dep*^ ECs restored their barrier responsiveness to thrombin (Fig. 8g).

### Zdhhc21^dep/dep^ reduces PLCβ1 palmitoylation and signalling

**Figure 9:**
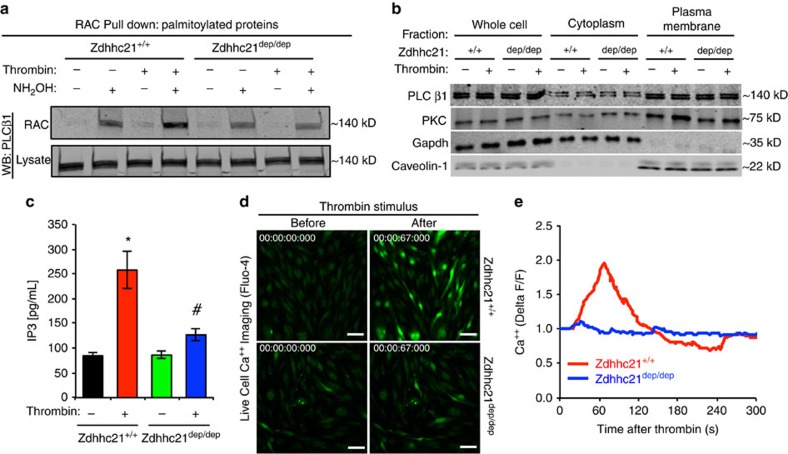
DHHC21 deficiency reduces inflammation-induced PLCβ1 palmitoylation and signaling activity. (**a**) The level of palmitoylated PLCβ1 in *Zdhhc21*^*+/+*^ and *Zdhhc21*^*dep/dep*^ ECs with or without thrombin (10 U ml^−1^) was determined using resin-assisted capture. Palmitoylated proteins in whole cell lysate were isolated by thiol-reactive sepharose resin in the presence of hydroxylamine (NH_2_OH) and then used for immunoblotting with anti-PLCβ1. Blot is representative of four independent experiments. (**b**) Membranous and cytosolic fractionation assay showing the subcellular distribution of PLCβ1 and its downstream signal PKC in *Zdhhc21*^*+/+*^ and *Zdhhc21*^*dep/dep*^ ECs with or without thrombin (10 U ml^−1^). Images are representatives of three independent experiments. Gapdh and caveolin-1 were used as internal loading controls (Gapdh for whole cell and cytoplasm fraction, caveolin-1 for plasma membrane fraction). (**c**) IP3 levels indicative of PLCβ1 signaling activity in *Zdhhc21*^*+/+*^ and *Zdhhc21*^*dep/dep*^ ECs subjected to thrombin (10 U ml^−1^) stimulation. Bar graph represents mean±s.e.m. from three independent experiments. *P<0.05 versus *Zdhhc21*^*+/+*^ EC+control, ^#^*P*<0.05 versus *Zdhhc21*^*+/+*^ EC+thrombin. (**d**,**e**) Intracellular calcium spike indicative of PLCβ1 activation in ECs isolated from *Zdhhc21*^*+/+*^ or *Zdhhc21*^*dep/dep*^. Data represents three independent experiments. (**d**) Representative images of live EC monolayers labelled with the calcium indicator Fluo-4 before and after thrombin stimulation. (**e**) Representative tracings of intracellular calcium concentration.

Since PLCβ1 was identified as a specific palmitoylation target involved in inflammation, we hypothesized that PLCβ1 might serve as the substrate of DHHC21 that accounts for inflammation-mediated endothelial injuries. The effects of DHHC21 function on the PLCβ1 palmitoylation during inflammation were determined by resin-assisted capture, an assay that allows palmitoylated proteins to be cleaved by hydroxylamine (NH_2_OH) and captured with thiol-reactive Sepharose resin. Our data demonstrated that thrombin induced a detectable increase in PLCβ1 palmitoylation in *Zdhhc21*^*+/+*^ ECs, which was attenuated in *Zdhhc21*^*dep/dep*^ ECs (Fig. 9a). Given that palmitic acid attachment can alter protein hydrophobicity and membrane association^23^, we then compared membrane localization of PLCβ1 between *Zdhhc21*^*+/+*^ and *Zdhhc21*^*dep/dep*^ ECs. However, no significant difference of PLCβ1 plasma membrane localization was found between these two cells under baseline or inflammatory conditions (Fig. 9b).

Thrombin-induced increased membrane localization of PKC, a downstream effector of PLCβ1 signalling known to translocate to cell membranes upon activation, was inhibited in DHHC21 deficient ECs (Fig. 9b), supporting the involvement of DHHC21-induced PLCβ1 palmitoylation in PLCβ1 signalling activation. Two other effectors of PLCβ1, namely IP3 and intracellular calcium, were also evaluated as the indication of PLCβ1 downstream signalling activation. The level of IP3 was increased by thrombin stimulation, which was greatly attenuated in *Zdhhc21*^*dep/dep*^ ECs (Fig. 9c). Consistently, thrombin-elicited intracellular calcium spike was diminished in *Zdhhc21*^*dep/dep*^ MLMVECs compared with *Zdhhc21*^*+/+*^ ECs (Fig. 9d,e).

### DHHC21-PLCβ1 pathway in endothelial barrier dysfunction

**Figure 10:**
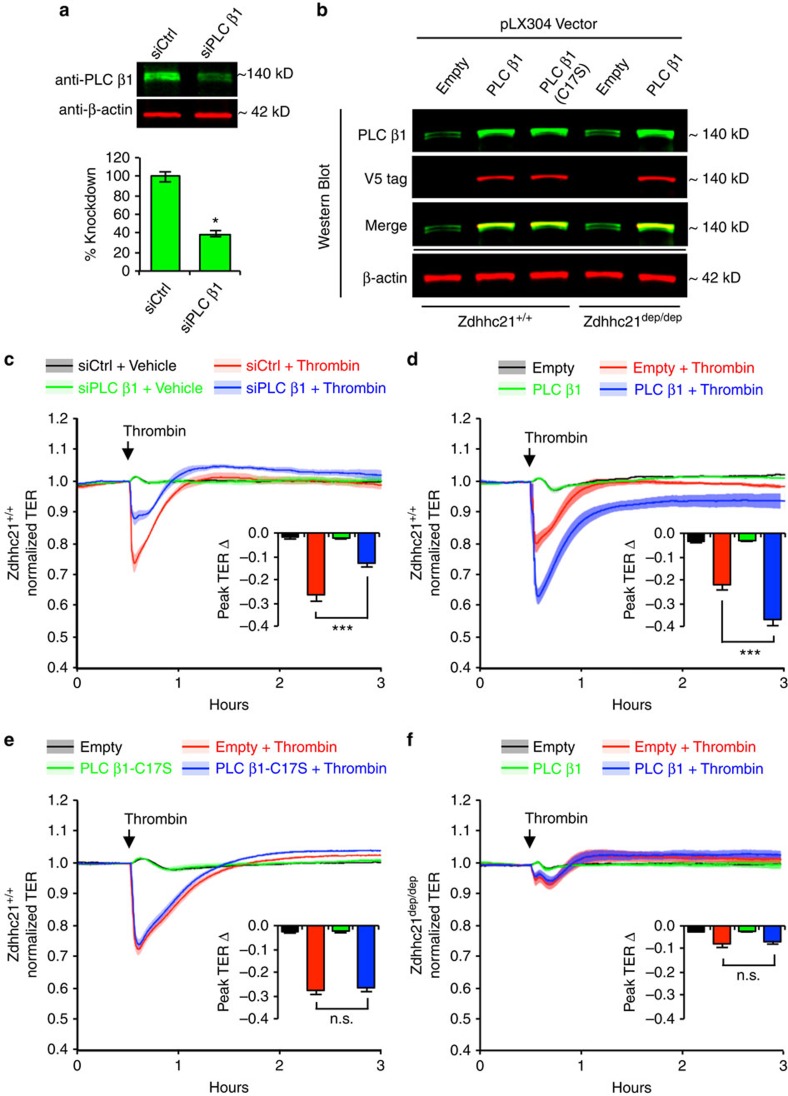
DHHC21-mediated PLCβ1 palmitoylation contributes to barrier dysfunction in inflammation. Molecular manipulations of DHHC21 or PLCβ1 via knockdown and site-specific mutagenesis indicate the involvement of DHHC21-PLCβ1 palmitoylation in endothelial barrier response. (**a**) Verification of PLCβ1 siRNA knockdown by Western blotting. β-actin serves as loading control. Results represent mean±s.e.m. from three independent experiments. *P<0.05 versus control siRNA. (**b**) Verification of *wild-type* PLCβ1 or C17S mutant PLCβ1 (V5 tagged) overexpression in *Zdhhc21*^*+/+*^ or *Zdhhc21*^*dep/dep*^ECs. PLCβ1 C17S mutant was created by site-specific mutagenesis at Cys17, the highly predicted palmitoylation site. Empty pLX304 vector serves as vector control. β-actin serves as loading control. (**c**) Dynamic recording of TER responses to thrombin (10 U ml^−1^) in PLCβ1 knockdown ECs. Solid line tracing represents the mean resistance, and shadow represents±s.e.m. Embedded bar graph shows peak TER changes and presented as mean±s.e.m. from three independent experiments. ***P<0.001. (**d**) The effect of overexpressing *wild-type* PLCβ1 on TER responses to thrombin (10 U ml^−1^) in *Zdhhc21*^*+/+*^ ECs. Solid line tracing represents the mean resistance, and shadow represents±s.e.m. Embedded bar graph shows peak TER value changes. Bar graph represents mean±s.e.m. from three independent experiments. ***P<0.001. (**e**) The effect of overexpressing C17S mutant PLCβ1 (palmitoylation impaired) on TER responses to thrombin (10 U ml^−1^) in *Zdhhc21*^*+/+*^ ECs. Solid line tracing represents the mean resistance, and shadow represents s.e.m. Embedded bar graph shows peak TER value changes. Bar graph represents mean±s.e.m. from three independent experiments. *n.s.*=not significant. (**f**) The effect of overexpressing wild-type PLCβ1 on TER responses to thrombin (10 U ml^−1^) in *Zdhhc21*^*dep/dep*^ ECs. Solid line tracing represents the mean resistance, and shadow represents s.e.m. Embedded bar graph shows peak TER changes. Bar graph represents mean±s.e.m. from three independent experiments. *n.s.*=not significant.

The critical role of PLCβ1 in mediating endothelial dysfunction is supported by the data that knockdown of PLCβ1 in *Zdhhc21*^*+/+*^ ECs led to a significantly attenuated TER response to thrombin (Fig. 10a,c). Furthermore, in an *in silico* analysis using the CSS palm palmitoylation algorithm^38^, only cysteine residue 17 of PLCβ1, with a high CSS-palm score of 27, was above the medium threshold and predicted to be palmitoylated. We thus created a mutant PLCβ1 by replacing Cys17 with serine to disable palmitoylation at this site. Comparative analyses were performed in *Zdhhc21*^*+/+*^ or *Zdhhc21*^*dep/dep*^ ECs overexpressing the mutant or *wild-type* PLCβ1 (Fig. 10b,d–f). The results indicated that overexpression of *wild-type* PLCβ1 in *Zdhhc21*^*+/+*^ ECs augmented TER reduction upon thrombin stimulation (Fig. 10d), whereas overexpression of C17S mutant PLCβ1 exerted no additional barrier dysfunction (Fig. 10e). Moreover, overexpression of *wild-type* PLCβ1 in *Zdhhc21*^*dep/dep*^ ECs failed to augment thrombin-induced barrier dysfunction (Fig. 10f). These data suggested the key role of DHHC21-catalysed PLCβ1 palmitoylation in mediating inflammation-induced endothelial barrier dysfunction.

## Discussion

The present study demonstrates a novel function of palmitoyl acyltransferases in mediating endothelial dysfunction during inflammation. We employed multiple *in vivo*, *ex vivo* and *in vitro* models relevant to SIRS, including trauma, endotoxemia and inflammatory stimuli. Our data reveal several new findings: (1) PAT inhibition attenuates inflammation-induced endothelial barrier dysfunction, microvascular leakage and leucocyte adhesion; (2) the cellular processes underlying PAT-induced injury involve endothelial cell–cell junction hyperpermeability and increased expression of ICAM-1; (3) 11 DHHC-PATs are detected to be expressed in vascular endothelium: DHHC2, 4, 5, 8, 9, 12, 13, 16, 17, 20 and 21, of which DHHC5 and 21 are required for barrier response to inflammatory stimulation; (4) mice with DHHC21 function deficiency are resistant to plasma leakage and leucocyte infiltration; they also display attenuated lung pathology and improved survival following traumatic or septic challenge; (5) DHHC21 deficiency confers protection against inflammatory injury via an endothelial-dependent pathway; and (6) DHHC21-mediated PLCβ1 palmitoylation and activation of its downstream signalling play an important role in mediating endothelial dysfunction during inflammation.

Remarkable heterogeneity exists in PATs with respect to their tissue/cell distributions and N- or C-terminal extensions^23^,^36^. Comparing mRNAs from multiple tissues, Ohno et al. showed that *Zdhhcs 4*, *5*, *7*, *8*, *10*, *12*, *13*, *17* and *22* were ubiquitously expressed, while others were highly tissue-specific^36^. For example, *Zdhhc11* was found only in testis, *Zdhhc19* in testis, thymus and small intestine, and *Zdhhc21* was found in multiple tissues including blood vessels, but is absent in leucocytes^33^,^36^. Within this context, although *Zdhhcs 2, 3, 7, 8* and *21* have been identified in human umbilical vein endothelial cells^39^, our experiments are the first to screen endothelial cells from different vascular beds for expression of all 24 *Zdhhcs*. We identified the presence of *Zdhhcs 2, 4, 5, 8, 9, 12, 13, 16, 17, 20* and *21* in mouse lung microvascular endothelial cells, cells frequently implicated in acute systemic inflammation and commonly used as a clinically relevant cell model. While the absence of *Zdhhcs 3* and *7* in our findings is not in perfect agreement with a previous report^39^, the discrepancy could be attributed to organ or species heterogeneity. Interestingly, *Zdhhc21* appears to be ubiquitous among endothelial cells from different vascular beds and between species, supporting its common involvement in the circulatory system. This is important because different DHHC-PATs may contribute to different cellular or pathological processes. For example, DHHC8 has been linked to schizophrenia^30^, DHHC9 to intellectual disability^40^, DHHC17 to type 1 diabetes and Huntington's disease^41^, and DHHC2, 9, 11 and 17 to cancer^42^. We believe our study is the first to demonstrate the pathophysiological significance of DHHC21 in inflammatory injury.

Proteins spanning from signalling molecules to membrane proteins have been shown, or predicted by proteomics analyses, to be palmitoylated in response to extracellular or intracellular stimulation^23^,^43^. Unlike other fatty acid modifications, S-palmitoylation is reversible as depalmitoylation can be catalysed by acyl protein thioesterases (APTs) 20. The multi-functionality and reversibility of palmitoylation has attracted rising interests in drug research. Inhibitors of palmitoylating enzymes have been developed and tested as therapies for cancer and autoimmune disease. Among the PAT inhibitors available in the market, 2-BP is best studied and most commonly used because of its relative selectivity and high potency toward PATs with minimal toxicity^44^,^45^,^46^. Cell culture experiments with 2-BP demonstrated considerable tolerability in endothelial cells, while 2-BP simultaneously prevented glucose-mediated toxicity 47. There are also reports on its short-term use as a tracer for *in vivo* experiments 48. In our study, we did not detect any obvious morphological or functional changes in endothelial cells incubated with 2-BP, and administration of the drug in animals did not cause mortality or significant abnormalities in circulatory function. In view of its potent protective effect on endothelial barriers without obvious adverse actions *in vivo*, the drug may have therapeutic potential as an anti-inflammation adjuvant. However, we do acknowledge the limitation of the pharmacological approach, as 2-BP might exert off-target effects that are not specific to vascular tissues. The drug could also affect other signalling processes than palmitoylation, or inhibit enzymes such as APTs and PPARγ 49. Although we conducted time-course and dose-response studies with 2-BP and compared its effects with other palmitoylation-altering agents including 2-hydroxymyristate and palmostatin-B, further studies are warranted to establish the pharmacokinetics and pharmacodynamics of these drugs under specific disease conditions. Nevertheless, the primary purpose of the current study was to provide proof-of-principle for the involvement of PATs in inflammatory signal transduction.

A particularly novel aspect of our study is the demonstration that DHHC21 function deficiency confers protection against endothelial injury during inflammation. The *Zdhhc21*^*dep/dep*^ is a genetically altered mouse model resulting from 3-base pair deletion in the *Zdhhc21* exon rendering its loss-of-function in catalysing palmitoylation. Compared with knockouts, this model offers a unique opportunity to specifically study its enzymatic activity so that pharmacological or molecular tools can be designed to manipulate its function without demolishing its expression. The mouse phenotype is characterized by depilation and heavily pigmented greasy skin 37. In our experiments, these mice were born at a normal gestational rate, were weaned into adulthood with no complications, and did not display obvious abnormalities in their basal cardiopulmonary or microcirculatory function. A more recent phenotypic characterization revealed attenuated α1 adrenergic-dependent vasomotor reactivity to phenylephrine and transient hypotension; however, no fluid imbalance, renal impairment or gross histological differences in the heart or kidney were found 33. Importantly, endothelial-dependent vasodilation was unaffected, making the *Zdhhc21*^*dep/dep*^ model advantageous for studying barrier-specific regulation of fluid dynamics with minimized confounding variables from hemodynamics. We chose to focus on *Zdhhc21*^*dep/dep*^ in this study because DHHC21 was one of the only two DHHCs required for endothelial hyperpermeability, as verified in our functional screening analysis using individual gene knockdown. Further, the absence of DHHC21 in leucocytes^36^ serves as a tool for distinguishing endothelial-dependent pathways in barrier regulation and leucocyte recruitment, processes regulated by molecules present in both cell types. It is noteworthy that DHHC5 was also identified to be present in endothelial cells and silencing its gene was able to attenuate endothelial barrier response. However, DHHC5 is ubiquitously expressed in circulating and parenchymal cells^36^, and relatively limited information is accessible regarding the cardiovascular phenotypes of *Zdhhc5* knockout mice.

The precise molecular mechanisms by which DHHC21 loss-of-function confers protection against aberrant endothelial activation require further studies. Given the recent report about low blood pressure in *Zdhhc21*^*dep/dep*^ mice^33^, we do not rule out the possibility that the observed protection was related to limited access of inflammatory mediators to important tissues under a relatively low perfusion pressure. However, our *in vitro* experiments using cells isolated from these mice suggest a direct effect of DHHC21 on endothelial junction and ICAM-1 expression. This is further supported by the fact that silencing DHHC21 produced similar protection as DHHC21 function deficiency. While the barrier effect of gene silencing appears small, it could be due to some residual expression of the enzyme. Alternatively, these relatively modest molecular reactions could translate into a significant phenotypic change *in vivo* altering microvascular transport and leucocyte recruitment in important organs. Endothelial barrier opening can further promote leucocyte transmigration thereby exacerbating inflammatory injury. Within this context, our imaging analysis of endothelial junctions showed that VE-cadherin staining became thinner and disrupted upon inflammatory stimulation, consistent with the notion that both junction protein degradation and conformational changes contribute to paracellular hyperpermeability.

The role of DHHC21 in regulating PLCβ1 palmitoylation and activity is supported by the data that DHHC21 deficiency attenuates inflammation-stimulated PLCβ1 palmitoylation coupled with activation of its downstream signalling as indicated by IP3 production, intracellular calcium spike and PKC membrane translocation. Moreover, endothelial cells overexpressing wild-type PLCβ1 displayed significantly augmented barrier dysfunction response to stimulation, whereas DHHC21 deficient cells or cells overexpressing PLCβ1 C17 mutant did not show such a phenotype, further indicating PLCβ1 palmitoylation as a target of DHHC21. Consistent with these mechanistic experiments, our proteomics analysis combining ABE with mass spectrometry identified a number of endothelial proteins undergoing palmitoylation upon stimulation by SIRS plasma, including PLCs and proteins related to the GPCR signalling pathway. How palmitoylation activates these signalling molecules remains an interesting question. Since palmitic acid attachment can alter the hydrophobicity of a protein thereby increasing its association with cell membranes^23^ where several DHHCs are located^20^,^24^,^29^,^36^, we initially hypothesized that palmitoylation might promote PLCβ1 recruitment to the cell membrane. To our surprise, however, we did not observe any significant difference of PLCβ1 distribution in the cytosolic and membranous fractions of endothelial cells, regardless of inflammatory states or the presence of DHHC21 function. In contrast, the membrane localization of PKC, a downstream effector of PLC signalling known to translocate to cell membrane upon activation, was increased after stimulation and this effect was attenuated in DHHC21 deficient cells. The PKC activation response correlates with those of other PLCβ1 downstream effectors such as IP3 and calcium, supporting a common role of PLCβ1 palmitoylation at the upstream of the signalling pathway. Thus, we propose that in response to inflammatory mediators or GPCR ligands (histamine, thrombin), endothelial signalling proteins downstream of their receptors (PLCβ1) undergo DHHC21-catalysed palmitoylation rendering enhanced signalling activities. Similar to kinases, DHHC21 may activate PLCβ1 by altering its enzymatic activity, molecular conformation, intracellular location, or ability to interact with other molecules. The possibility of PATs acting as kinase-like factors is supported by the similarity of palmitoylation-depalmitoylation and phosphorylation-dephosphorylation with respect to their reversible kinetics and rapid nature of reactions. The molecular mechanisms by which PLC activation induces endothelial cell contraction and paracellular permeability have been extensively investigated in our lab and others^18^,^50^,^51^,^52^. The downstream effectors of PLC (PKC, IP3 and calcium) and their interactions with cell–cell adhesion molecules including ICAM-1 are discussed in more detail elsewhere^53^,^54^,^55^,^56^.

In addition to the PLC cascade, other signalling or structural molecules may be involved in palmitoylation-mediated endothelial responses. In particular, several endothelial palmitoylproteins have been identified as direct substrates of DHHC21^39^,^57^,^58^, including platelet endothelial cell adhesion molecule (PECAM-1), eNOS, caveolin-1, SOD1 and Fyn, many of which have been implicated in endothelial inflammation^51^. For example, PECAM-1 facilitates leucocyte transendothelial migration, Fyn mediates dissociation of endothelial cell–cell junctions, and eNOS-derived NO increases vascular permeability^50^,^59^,^60^. In a recent study, DHHC21 knockdown resulted in decreased eNOS palmitoylation and impaired NO release^39^. Another study showed that inactivation of fatty-acid synthase caused endothelial barrier dysfunction via decreased eNOS palmitoylation^61^. The discrepant findings from these two studies might result from different experimental conditions used to manipulate palmitoylation; in particular, fatty-acid synthase is not a PAT but a multifunctional enzyme associated with palmitate production. Overall, the limited availability of PAT knockout models, along with the well-recognized technical difficulties in quantification of DHHC-specific activities, contributes to the paucity of information regarding palmitoylation substrates and functions. Thus, further work elucidating the full spectrum of quiescent and inflammation-induced DHHC21 targets in vascular endothelium would advance the pathophysiology of SIRS, while facilitating cell-targeting drug development. In this regard, our novel findings build an important foundation for future studies in this rapidly expanding field.

In summary, the present study demonstrates that DHHC21-PAT plays a critical role in mediating endothelial dysfunction during inflammation. The underlying mechanisms involve endothelial junction hyperpermeability and increased ICAM-1 expression mediated by palmitoylation of endothelial signalling molecules including PLCβ1. Targeting specific PATs, especially DHHC21, may serve as an effective therapeutic tool for treating vascular diseases or inflammatory injury.

## Methods

### Reagents and supplies

A complete list of reagents, supplies, company information and catalogue numbers can be found in Supplementary Tables 2–5.

### Animals

All rats used in these studies were male Sprague–Dawley (SD) purchased from Harlan Laboratories weighing 300–350 g. All mice (20–30 g) used in these studies were either *wild-type* (*Zdhhc21*^*+/+*^) or homozygous depilated mice that contain a spontaneous mutation in the *Zdhhc21* gene, *Zdhhc21*^*dep/dep*^ mice. These mice were purchased from Jackson Laboratory 37. To confirm murine genotype, tissue samples were sent for sequencing (Genewiz, Inc., NJ, USA). The primers for sequencing *Zdhhc21*^*dep/dep*^ were: AGCTGACTGAAGGGCACC (F); AAAACCTGTAACGCATTTCCA (R). Animals were maintained under a 12/12-hour light/dark cycle with food and water ad libitum. Animals were randomly allocated to experiment groups using random number table by investigators. The information regarding the animal strain, age and sex are provided in Supplementary Table 6. All surgical and experimental procedures involving animals were approved by the University of South Florida Institutional Animal Care and Use Committee.

### Induction of Systemic Inflammatory Response Syndrome (SIRS)

SIRS was induced in animals subjected to thermal trauma (sterile inflammation) or septic injury (infectious inflammation). Thermal injury was performed based on a modified Walker and Mason burn model 62. Briefly, mice or rats were anesthetized with 1.75 × 10^3^ mg kg^−1^ urethane. The sides and back of the anesthetized animals were shaved carefully. Hot water at 100 °C was applied for 9 s on the dorsal surface of the mouse to induce a full-thickness scald burn covering 25% total body surface area (TBSA) (40% TBSA in rats). For resuscitation 37 °C Lactated Ringer's (LR) fluid (4 ml kg^−1^ × per cent TBSA, according to Parkland formula^62^) was injected subcutaneously on the dorsum of the animal immediately post burn. For sham groups, animals were subjected to the same treatment except the water temperature was 37 °C. To induce septic SIRS, ultra-pure *E. coli* lipopolysaccharide (LPS) was freshly dissolved in LR. Rats or mice were injected (IP) with LPS at the dose of 10 mg kg^−1^. Control rats or mice received LR alone. For thermal injury survival studies, SIRS was induced as mentioned above, except isoflurane was used for anaesthesia during induction. All animals were monitored 48 h post-induction. For LPS survival studies, mice were monitored 72 h post-induction.

### Lung injury score survey

SIRS induction was performed as above. After 24 h, mice were anesthetized, exsanguinated by transcardial perfusion with PBS, and perfusion fixed with 4% paraformaldehyde. The lungs were carefully excised, post-fixed in 10% neutral buffered formalin for 48 h, and sent to HistoWiz Inc. for processing, embedding, sectioning, staining, and imaging. The lung injury was evaluated and scored by two blinded investigators based on the following histological features: (1) alveolar wall thickness, (2) exudate accumulation and (3) interstitial leucocyte infiltration^63^. The extent of injury for each parameter was graded from 0 to 3 based on the severity. A composite score was calculated for each mouse on a scale of 0–6.

### Intravital microscopy analysis of protein transvascular flux

Plasma protein flux across mesenteric microvessels was measured. Mice or rats were anesthetized with an intramuscular injection of urethane at 1.75 × 10^3^ mg kg^−1^. Cannulation of the left jugular vein was performed for IV infusion of drugs or solutions. Animals were restrained and body temperature was maintained at 37 °C with a heating pad (Fine Science Tools, North Vancouver, BC). The abdomen of the animal was shaved, a midline laparotomy was performed, and a section of mesentery from the proximal ileum was exteriorized over an optical stage for microscopic observation. To prevent the exteriorized mesentery from drying it was constantly superfused with 37 °C LR solution. Continuous IV infusion of LR at 0.04 ml min^−1^ kg^−1^ body weight was given to replenish fluid loss during the experiment. The mesenteric microcirculation was examined using a Nikon Eclipse E600FN Microscope under a × 10 working distance objective (Nikon Instruments Inc., NY, USA) equipped with a Cascade 512F digital camera (Photometrics, AZ, USA). To quantify transvascular flux of plasma proteins animals were given an IV bolus of fluorescein isothiocyanate conjugated bovine albumin (FITC-albumin) at 100 mg kg^−1^ (rats) or 15 mg kg^−1^ (mice), followed by continuous infusion at 0.15 mg kg^−1^ min^−1^ to maintain a constant plasma concentration. Postcapillary venules were selected for measurement of FITC-albumin flux. FITC-albumin leaking into extra-vascular space was accumulated over time. Fluorescent images were acquired every 5 min for 1 h and analysed using ImageJ (v1.48i; NIH, USA). Fluorescence intensity (*I*) was measured from windows positioned inside (*Ii*) and outside (*Io*) a selected venule. Time dependent FITC-albumin transvascular flux was calculated with the following equation: 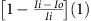. Mesenteric transvascular flux in SIRS and sham-SIRS was started 3 h after the thermal injury. 2 mg kg^−1^ 2-BP or vehicle was given IV 20 min before thermal injury. For histamine challenge in *Zdhhc21*^*+/+*^ versus *Zdhhc21*^*dep/dep*^ mice, baseline transvascular flux was obtained followed by exchange of 10 μM histamine in LR as the superfused buffer.

### Measurement of hydraulic conductivity

Microvascular permeability to water was determined using a modified Landis micro-occlusion technique that measures fluid filtration across unit areas of the microvascular wall under a set of controlled perfusion pressures. Briefly, rats were anesthetized with 1.75 × 10^3^ mg kg^−1^ intramuscular injection of urethane. The rat mesentery was exteriorized and superfused with LR solution at 37 °C. The microcirculation was observed through a CKX41 Inverted Microscope (Olympus, PA, USA) under a × 20 working distance objective, and images were recorded via a CoolSNAP ES^2^ video camera (Photometrics). A single postcapillary venule (30–50 μm in diameter) was cannulated with a micropipette containing 10 mg ml^−1^ BSA and red blood cells as markers. The micropipette was connected to a water manometer, allowing continuous flow of the perfusate under controlled intraluminal hydrostatic pressures. After equilibration, the downstream of the vessel was occluded with a glass rod. The initial transvascular flow per unit area of the vessel wall (*J*_v_/A) was measured. Hydraulic conductivity (*L*_p_) was calculated as the ratio of *J*_v_/A to perfusion pressure: *L*_p_=(*J*_v_/A)/P(2). 2-BP was administrated 30 min before histamine stimulation and continuously perfused until the end of the observation. Measurements were made for a 30-min period at 5-min intervals.

### Lung solute extravasation assays

Vascular permeability to solutes was assessed by measuring extravasation of the plasma albumin marker Evans blue (EB) and small molecule marker Sulfo–N-hydroxysuccinimide (NHS)–biotin. For EB extravasation, mice or rats received 160 mg kg^−1^ EB IV allowing circulation for 30 min. Circulation was replaced with LR fluid by performing transcardiac perfusion. To determine EB concentration, the flushed lungs were removed and homogenized in 1 ml PBS. Lung homogenates were incubated with 2 ml formamide (Sigma, F9037) at 60 °C for 18 h to extract EB and centrifuged at 5000*g* for 30 min. The concentration of EB in the supernatant was detected by a dual wavelength spectrophotometric method at 620 and 740 nm using the following formula: E_620_(corrected)=E_620_−(1.426 × E_740_+0.030) (3). A standard solution of EB in formamide was freshly made at the concentrations of 0, 1, 2, 4, 8, 16, 32, 64 ng ml^−1^, and the results are presented as ng of EB per mL by comparing with the standard curve. To visualize EB extravasation, lungs were flushed, perfusion-fixed, and post-fixed overnight in 4% paraformaldehyde (PFA, ACROS, 41678). Following PBS washes, the entire left lobe was imaged for EB autofluorescence at 700 nm with an infrared imaging system (Odyssey CLx; Li-Cor) at a resolution of 21 μm. The same lobe was then embedded in 3% agarose and sliced into 1 mm transverse sections and imaged as above. The images were pseudo-colored with a heat-map colour scheme to highlight inter-sample signal intensity. For small solute permeability, sulfo–N-hydroxysuccinimide–biotin extravasation was performed^64^. Briefly, sham and SIRS rats were perfused with 30 ml 0.3 mg ml^−1^ of the low molecular weight lysine-reactive biotinylation reagent, followed by perfusion fixation with 2% PFA, lung-excision and post-fixation in 2% PFA. Lung slices of 1 mm and 100–150 μm vibratome sections were incubated overnight in streptavidin conjugated with either IRdye-800 or Texas-Red (Vector, SA-5006) diluted in PBSTC (PBS+0.5% Triton-X100+0.1 mM CaCl_2_) with 10% normal goat serum, and washed for 6 h in PBSTC. Slices were imaged as above in the Odyssey CLx at 800 nm. Vibratome sections were mounted in vectashield with DAPI and confocal micrographs were obtained with Olympus FV1000 MPE laser scanning microscope.

### Intravital microscopic measurement of leucocyte dynamics

Leucocyte-endothelial interactions were analysed in mesenteric and ear microcirculation. Rat LPS-SIRS was induced as above with or without 2-BP (2 mg kg^−1^; IV). after induction of 4 h, rats were anesthetized with 1.75 × 10^3^ mg kg^−1^ urethane. Rats were prepared for microscopic observation as mentioned above. Briefly, a loop of proximal ileum was exteriorized over an optical stage after midline laparotomy. Constant dripping of LR solution was applied to prevent the exteriorized mesentery from drying. Body temperature was maintained as described above. Leucocytes were fluorescently labelled with acridine orange (0.25–0.5 ml; 1.65 × 10^−4^ M) and visualized in post-capillary venules using a Zeiss Axiovert 200 inverted microscope under a × 10 Zeiss objective equipped with a Photometrics Cool Snap HQ2 camera (Photometrics, AZ, USA). The interaction of leucocytes with venular wall was recorded for 60 s in at least 3 venules per rat. Four parameters were measured to evaluate leucocyte interaction with mesenteric venular wall^65^: slow-rolling flux, slow-rolling flux fraction, leucocyte rolling velocity and leucocyte adhesion. Rolling leucocytes were defined as leucocytes moving at a significantly reduced velocity compared with centerline blood flow velocity. Slow-rolling flux was quantified as the number of leucocytes rolling at the velocity of <5 μm s^−1^ in the observed venule within 60 s; slow-rolling flux fraction was as the percentage of the number of slow-rolling leucocytes to the total number of rolling leucocytes; leucocyte rolling velocity was determined as the average velocity of all rolling leucocytes in the observed venule. Leucocyte adhesion was measured by counting the number of adherent leucocytes (no visible movement for more than 20 s) per 10^5^ μm^2^. Video analyses and measurements were performed by an independent investigator blinded to experimental design. In separate groups of mice, leucocyte-endothelial interactions were recorded 4 h post-SIRS induction as above, except microscopic observations were made in venules of mouse ears secured to an optical stage.

### Primary endothelial cell culture

Individual reagents, company information and catalogue numbers can be found in Supplementary Table 2. Cells were seeded in 0.1% gelatin coated culture flasks (Corning, NY, USA). Human umbilical vein endothelial cells (HUVECs, Lonza, MD, USA) were grown in Endothelial Cell Basal Medium supplemented with EGM-2 MV Bulletkits (Lonza, MD, USA). Primary mouse lung microvascular endothelial cells (MLMVECs, Cell Biologics, Inc.) were cultured in Mouse Endothelial Cell Medium with Supplement Kit (Cell Biologics, Inc., IL, USA). All cells were incubated in a 5% CO_2_ humidified incubator at 37 °C to reach confluence 2–3 days before experiments. Primary MLMVEC were commercially isolated (Cell Biologics, Inc.) from 5 to 8 day old *Zdhhc21*^*+/+*^ and *Zdhhc21*^*dep/dep*^ mice.

### Leucocyte isolation

Whole blood from mice was obtained by cardiac puncture, and layered over a Histopaque 1077/1119 gradient. After centrifugation at 700*g* for 30 min, RBC lysis was performed, and white blood cells were rinsed and collected for use. Whole blood from humans was obtained commercially (AllCells, LLC). Leucocytes were isolated by mixing the blood with 6% dextran in normal saline and incubated for 1 h at room temperature. The supernatant was collected and centrifuged at 200*g* for 12 min. Leucocytes were washed, RBC lysis was performed, and leucocytes were rinsed and collected for use.

### Endothelial barrier function assays *In vitro*

The barrier function of cultured endothelial cell monolayers was determined by measuring TER, an indicator of cell–cell adhesive barrier resistance, using an electric cell-substrate impedance sensing system (Applied Biophysics, Troy, NY) 66. Electric cell-substrate impedance sensing tracings are expressed as TER normalized to baseline in untreated conditions. TER changes were recorded and submitted for statistical analyses of peak changes. In separate experiments, transendothelial flux of albumin was measured using a Transwell assay^66^. Briefly, 2 × 10^5^ MLMVECs isolated from either *Zdhhc21*^*+/+*^ and *Zdhhc21*^*dep/dep*^ mice were seeded in the upper ‘luminal' chamber of 0.4 μm Transwell inserts (Corning, MA, USA) that were coated with 0.1% gelatin and maintained in 24-well culture dishes. Media was maintained at a ratio of 100 μl in the luminal chamber and 670 μl in the lower ‘abluminal' chamber to insure negligible hydrostatic pressure. After culture for 2 days post-confluence, 20 μl of media from luminal chamber was replaced with 10 μl of media containing FITC-albumin (5 mg ml^−1^), followed by 10 μl of media containing thrombin (100 U ml^−1^) or vehicle control (0.1% BSA in PBS). After 30 min, samples were collected from both the luminal and abluminal chambers for fluorometry analysis. The concentration of FITC-albumin was determined using a standard curve, and the permeability coefficient of albumin (*P*_a_) was calculated as follows: *P*_a_=[*C*_A_/*t*] × [1/*A*] × [*V*/*C*_L_] (4), where [*C*_A_] is the abluminal FITC-albumin concentration, *t* is time (in seconds), *A* is the area of the membrane (in cm^2^), *V* is the volume of media in the abluminal chamber, and [*C*_L_] is the luminal FITC-albumin concentration.

### Leucocyte-endothelial cell adhesion assay

ECs were cultured to confluence on gelatin-coated coverslips. ECs were pre-stimulated for 4 h with vehicle control or 100 ng ml^−1^ IL-1β. After stimulation, ECs were rinsed three times with warm HBSS. Prior to endothelial-leucocyte incubations, ECs were labelled with CellMask Deep Red Stain, and leucocytes were labelled with CellTracker Green as per manufacturer's instructions. Leucocytes were incubated with ECs for 1 h. Before fixation (4% PFA for 10 min), non-adherent cells were washed three times with HBSS. Coverslips were mounted with Vectashield mounting medium containing DAPI. Images were acquired using Olympus FV1000 MPE laser-scanning microscope. The number of adherent leucocytes was counted and the results were presented as the number of adherent leucocytes per 10^5^ μm^2^.

### Immunocytochemistry

Immunocytochemistry (ICC) on confluent EC monolayers was performed per standard protocols. Briefly, after treatment with or without 1 h pretreatment of 2-BP, coverslips were fixed with 4% paraformaldehyde × 10 min, permeabilized with 0.05% Triton X-100 in PBS × 10 min, blocked with 5% BSA in PBST × 1 h and labelled with goat anti-VE-cadherin overnight at 4 °C. Secondary incubation for 1 h was done with donkey anti-goat AlexaFluor568. Cell-surface ICC for ICAM-1 was performed as mentioned, except that detergents were omitted from all solutions and secondary antibody was donkey anti-rabbit Alexa Fluor 488. ProLong Diamond mounting medium with DAPI was used for mounting coverslips. Fluorescent images were captured with Olympus FV1000 Olympus MPE laser scanning microscope. Images were analysed via Imaris software (Bitplane). Briefly, the intensity threshold of VE-cadherin channel was set for each ICC image to label all the VE-cadherin positive regions (region of interest, ROI). The number of ROI was then counted without bias using Imaris. Continuous VE-cadherin staining corresponds with a lower number of ROI, whereas discontinuous staining corresponds to a higher number of ROI. The results of VE-cadherin discontinuity were presented as the number of ROI counted via Imaris.

### On-cell western assay for ICAM-1 cell surface expression

On-Cell Western Assays were performed per the manufacturer's (Li-Cor) instructions and standardized protocols. Briefly, ECs were grown to confluence in gelatin-coated 96-well plates. After treatment, ECs were fixed and immunolabeled as per the ICC protocol for ICAM-1 surface staining. Total cell staining (CellTag) for normalization was performed during secondary antibody incubations. Images were scanned and analysed with Li-Cor Odyssey Clx.

### Real-time PCR

Total RNA from MLMVECs was extracted using RNAzol RT per manufacturer's instructions. RNA purity and concentration was quantified with an Agilent 2100 Bioanalyzer (Agilent Technologies, TX, USA). Total RNA of 1 μg was used per 20 μL of iScript cDNA Synthesis Kit. Prime PCR Reverse Transcriptase control was used during cDNA synthesis per the manufacturer's instructions. Wet-lab validated gene-specific PCR primers from Bio-Rad were used to analyse mRNA expression with PCR. Qualitative PCR amplifications were performed in a CFX Connect thermocycler with the following programme: 1 step of 2 min at 95 °C, 35 cycles of 15 s at 95 °C, 30 s at 60 °C and 5 s at 72 °C, and the final step of 7 min at 72 °C. PCR amplified products were electrophoresed in 4–20% Criterion precast polyacrylamide TBE gels ∼90 min at 150 V and stained with Syto 60 for visualization on Odyssey CLx.

### Acyl-Biotin exchange

Acyl-Biotin Exchange (ABE) was performed per recently developed protocols with modifications^57^. HUVECs were treated with inflammatory stimuli and then lysed with lysis buffer (50 mM Tris, 5 mM EDTA, 150 mM NaCl, 1% NP-40, 10% glycerol, 5 mM MgCl_2_, a tablet of complete ULTRA protease inhibitor, pH 7.4) on ice. Lysates were centrifuged at 13,000*g* for 10 min at 4 °C. Supernatants were collected and protein concentrations were determined using BCA assay. Proteins were precipitated using several volumes of ice-cold acetone for 30 min at −20 °C, collected by centrifugation and resuspended in 37 °C 4SB buffer (4% SDS, 5 mM EDTA, 50 mM Tris, pH 7.4). The solution was diluted to 1% SDS using lysis buffer. Methyl methanethiosulfonate (MMTS) was added to a final concentration of 20 mM and the solution was incubated for 30 min at 50 °C. Proteins were washed by three sequential rounds of precipitation in ice cold acetone followed by resuspension in 37 °C 4SB. Proteins were diluted to 1% SDS and split. Both samples were treated with 1 mM biotin HPDP for 60 min at room temperature (RT). One sample was then treated with 1 M hydroxylamine (HA, pH 7.4). The other half was treated with Tris buffer (pH 7.4) as control. Proteins were washed three times with acetone and resuspended in 4SB buffer. The solution was diluted to 1 ml with dilution buffer (50 mM Tris, 150 mM NaCl, 5 mM EDTA, 0.2% Triton-X 100, pH 7.4). To each tube 100 μl prewashed streptavidin-conjugated agarose beads were added and rocked overnight at 4 °C. The beads were washed three times with dilution buffer and the captured proteins were eluted using 100 μl dilution buffer with 1% β-mercaptoethanol. BCA assay was performed again, and 4 × sample buffer with 2.5% β-mercaptoethanol was added to proteins. Samples were loaded into gels for mass spectrometry mail-out and/or subsequent western blotting per standard protocols.

### Click chemistry

Confluent HUVEC monolayers were incubated with 100 μM Alk-C16 overnight to metabolically label palmitoylated proteins. Alk-C16 was prepared as follows: (1) dissolved in DMSO to the stock concentration of 50 mM, (2) diluted to a final concentration of 100 μM using EC culture medium with charcoal stripped FBS, (3) sonicated for 15 min at room temperature before use. Confocal microscopic imaging was then performed to analyse palmitoylated proteins 67. After metabolic labelling with Alk-C16, ECs grown on coverslips were then treated with 10 U ml^−1^ thrombin, 10 μM histamine or 20% SIRS plasma with or without 3 h pretreatment of 10 μM 2-BP. After washing with HBSS once, cells were fixed with pre-chilled methanol for 10 min in −20 °C. Cells were then permeabilized with 0.1% Triton X-100 in PBS for 5 min and washed with PBS six times. Click reaction solution was freshly made: 0.1 mM Oregon Green 488 azide (5 mM stock solution in DMSO), 1 mM Tris (2-carboxethyl) phosphine hydrochloride (TCEP, 50 mM freshly prepared stock solution in ddH_2_0), 0.1 mM tris[(1-benzyl-1H-1,2,3-triazol-4-yl)methyl]amine (TBTA, 10 mM stock solution in DMSO/tert-butanol (20%/80% V/V)) and 1 mM CuSO_4_ (50 mM stock solution in ddH_2_O) in ddH_2_O. Cells were treated with click reaction solution for 1 h in dark at room temperature, and then wash six times with PBS. ProLong Diamond mounting medium with DAPI was used for mounting coverslips. Images were acquired using Olympus FV1000 MPE laser-scanning microscope. The fluorescence intensity of palmitoylated proteins was analysed by Image J (v1.48i, NIH, USA).

### PLCβ1 palmitoylation assay

After metabolic labelling palmitoylated proteins, ECs were treated with 10 μM histamine. Cells were then lysed with 1 × RIPA buffer with a tablet of complete ULTRA protease inhibitor based on standardized protocol. To remove excess Alk-C16, samples were then subjected to chloroform-methanol protein precipitate where 4 volumes of methanol, 1 volume of chloroform and 3 volumes of ddH_2_O were sequentially added. Samples were then separated into two layers via centrifugation at 14,000*g* for 2 min with precipitated proteins located between the two layers. After removing the top layer, 4 volumes of methanol were added. Proteins were pelleted by centrifugation at 14,000*g* for 5 min. After removing supernatant, samples were air-dried for 5 min. Precipitated protein pellets were dissolved in 1% SDS and the protein concentrations were determined using BCA assay. Azide agarose resin was prepared according to manufacturer's instructions. Click chemistry solution (2 mM TCEP, 0.4 mM TBTA and 2 mM CuSO_4_) was freshly made before use. For each sample, 200 μL prepared agarose resin slurry, 800 μL protein sample and 1 ml Click chemistry solution were mixed thoroughly and incubated with end-over-end rotation for 20 h at 4 °C. Samples were then washed six times with washing buffer (100 mM Tris, 1% SDS, 250 mM NaCl, 5 mM EDTA, pH 8.0). After eluted by hydroxylamine (1 M, pH 7.0), proteins were precipitated and re-dissolved in 1 × protein loading buffer. Alternatively, palmitoylated proteins on agarose resin were eluted by 1 × protein loading buffer containing a high concentration of β-mercaptoethanol (1.43 M). The level of palmitoylated PLCβ1 in eluted samples was then determined with Western blotting.

### Resin-assisted capture

The level of palmitoylated protein was detected by resin-assisted capture^68^. Confluent MLMVECs were treated with 20 U ml^−1^ thrombin for 5 min and then lysed in lysis buffer (100 mM HEPES, 25 mM NaCl, 1 mM EDTA, 10 μM palmostatin B, protease inhibitors, pH 7.4). Cell lysate was then sonicated and incubated with blocking buffer (100 mM HEPES, 1 mM EDTA, 2.5% SDS, 6 μl ml^−1^ MMTs, pH 7.4) at 50 °C for 30 min with frequent vortexing. 4 volumes of cold acetone were added and incubated at −20 °C overnight to allow protein precipitation. Following centrifugation of the solution at 2,500*g* for 30 min, the protein pellet was washed with 70% cold acetone three times, and resuspended into binding buffer (100 mM HEPES, 1 mM EDTA, 1.0% SDS, pH 7.4). Protein quantification was performed and total protein amount for each group was normalized. The protein sample was split into two equal parts. Hydroxylamine (pH=7.4, final concentration of 0.2 M) and prewashed water-swollen thiopropyl sepharose 6B were added into one part. To the other part, an equal amount of NaCl and beads were added. The binding reaction of the beads and palmitoylated protein were carried out at room temperature for 4 h with constant rotation. Beads were washed five times with binding buffer, then eluted with 1 × sample buffer supplemented with 50 mM DTT at room temperature for 30 min. The solution mixture was heated to 95 °C for 5 min and centrifuged at 14,000*g* for 5 min. Supernatant was collected for SDS-PAGE.

### Membranous and cytosolic fractionation assay

Isolated MLMVECs were treated with thrombin (10 U ml^−1^) for 3 min. After washing three times with cold HBSS, ECs were lysed. Plasma membrane fraction of EC lysate was separated from cytosol fraction via Plasma Membrane Isolation Kit per manufacturer's instructions. Purified cellular fractions were then subject to Western blot.

### IP3 ELISA

HUVECs were treated with histamine (10 μM), and or 2-BP (100 μM) and immediately harvested, frozen and used for determining IP3 concentrations with an IP3 ELISA Kit per the manufacturer's instructions.

### Time-Lapse Ca^2+^ Imaging

Primary MLMVECs isolated from *wild-type* and *Zdhhc21*^*dep/dep*^ mice were grown to confluence on glass bottom culture dishes. Prior to imaging and thrombin stimulation, cells were prepared with the reagents from the Fluo-4 Calcium Imaging Kit per the manufacturer's instructions. Time-lapse images were acquired with 3i spinning disk confocal system (Intelligent Imaging Innovations) on an Olympus IX81 inverted microscope equipped with a stage-top microenvironmental chamber (Pathology Device) for constant 37 °C and 5% CO_2_. Time-lapse images were obtained with Olympus 20X UPLSAPO (NA 0.75) objective by acquiring images every second with Slidebook 6.0 software (Intelligent Imaging Innovations). To minimize spontaneous Ca^2+^ release due to exposure to the laser, cells were illuminated with 50% laser power and the fluorescence was captured with Evolve EMCCD (Photometrics) with a setting of 17-ms exposure and intensification mode. The relative fluorescence intensity fluctuation during the time-lapse was analysed by Slidebook 6.0.

### siRNA Transfection and gene transfer

All *Zdhhc* and PLCβ1 siRNA were purchased from Santa Cruz Biotech. pCMV6 empty vector, and ZDHHC21 plasmids were purchased from Origene; and pLX304 empty vector and PLCβ1 plasmids were purchased from Genecopoeia. MLMVECs grown to 90% confluence were trypsinized, pelleted, and resuspended in 100 μl of P5 Primary Cell 4D-Nucleofector^TM^ X with 1 μM siRNA or 2 μg plasmid. Cells were rapidly electroporated using the 4D-Nucleofector^TM^ System (Lonza, MD, USA) and plated in Endothelial cell complete medium (Cell Biologics) for experiments. Mutation at potential palmitoylation site of PLCβ1 was created by mutagenesis using as template the pLX304 with PLCβ1 transcript variant 1 cDNA clone purchased from Genecopoeia; and the Q5 Site-Directed Mutagenesis Kit with supplied 5-alpha competent cells purchased from New England BioLabs. PLCβ1 C17S mutagenesis primers (Fw: 5′ AAGCCCGTGTCCGTGTCCGAC 3′; Rev: 5′ GAGTTGCAAGGCGTGCAC 3′) were designed using the NEBaseChanger tool also provided by New England BioLabs. Successful mutation of C17S was confirmed by sequencing using (Fw: 5′ ACATCAATGGGCGTGGATAG 3′; Rev 5′ GGAAAGCCACGAGATTCAAATG 3′) designed with the PrimerQuest tool provided by Integrated DNA Technologies and Sanger sequencing of PCR product provided by Genewiz.

### Statistical analysis

All statistics were performed using GraphPad Prism (version 6.0f). See Supplementary Table 7 for a list of statistical tests used for all data analyses. Statistical tests were justified as appropriate. All data meet the normal distribution assumption. The variance is similar between groups that are being statistically compared. The sample size was derived from a priori power analysis based on the expected effect sizes observed in previous studies in our lab for each endpoint measurement and literature searches for commonly observed means and standard deviations between groups.

### Data availability

The authors declare that the data supporting the findings of this study are available within the article and its supplementary information files.

## Additional information

**How to cite this article:** Beard, R. S. *et al*. Palmitoyl acyltransferase DHHC21 mediates endothelial dysfunction in systemic inflammatory response syndrome. *Nat. Commun.* 7:12823 doi: 10.1038/ncomms12823 (2016).

## Supplementary Material

Supplementary Information Supplementary Figures 1-5 and Supplementary Tables 1-7.
