# Supplementary material for: Palmitoyl acyltransferase DHHC21 mediates endothelial dysfunction in systemic inflammatory response syndrome
Source: Nat Commun. 2016 Sep 22;7:12823. doi: 10.1038/ncomms12823 (PMC5036164; doi:10.1038/ncomms12823)
Supplement: Supplementary Information — Supplementary Figures 1-5 and Supplementary Tables 1-7. [file ncomms12823-s1.pdf]

Supplementary Figure 1

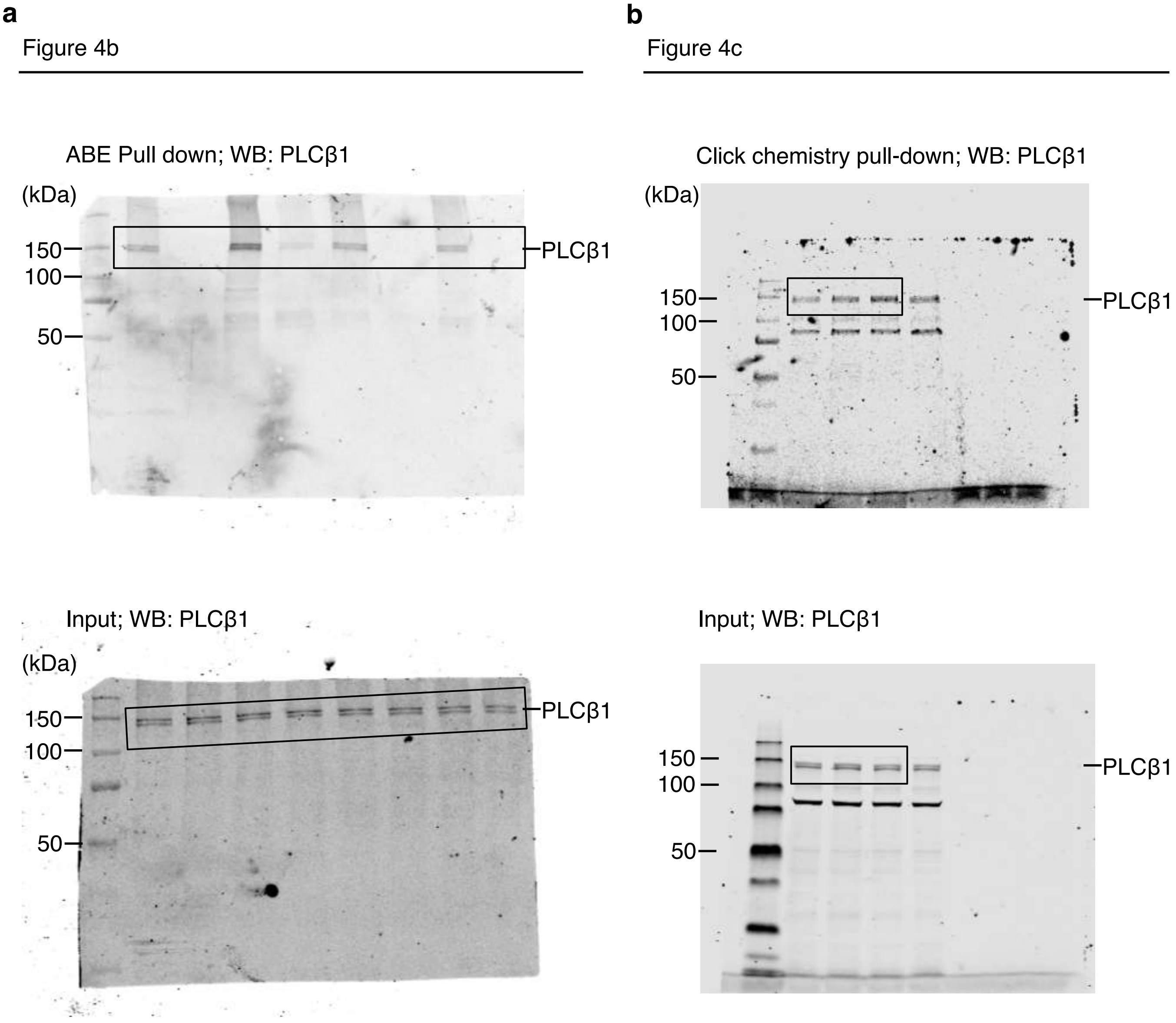

Supplementary Figure 1: Full-scanned images of western blots in main Figure 4. a**)** Full scans of blots in main Figure 4b. b**)** Full scans of blots in main Figure 4c.

Supplementary Figure 2

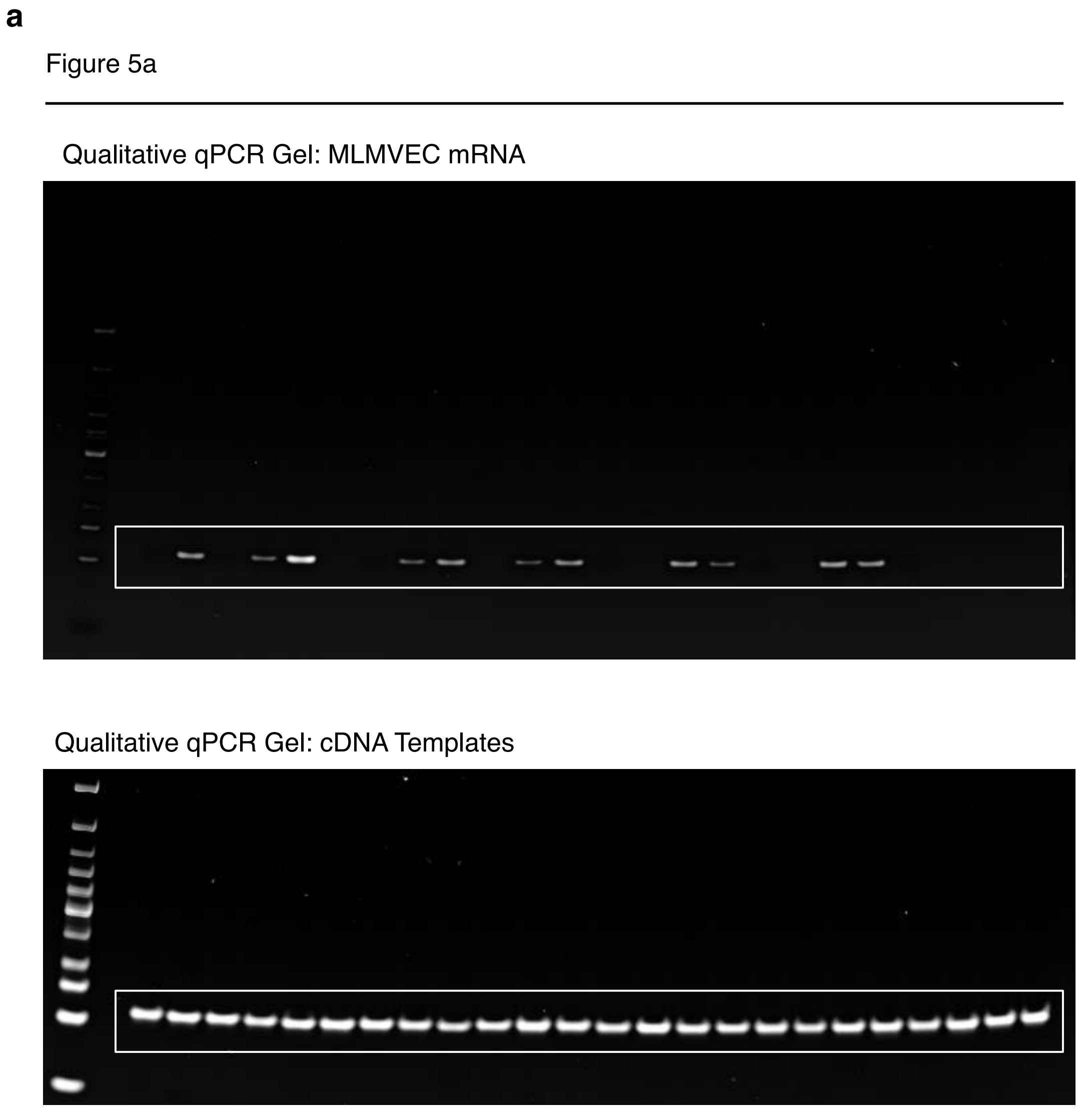

Supplementary Figure 2: Full-scanned images of gels in main Figure 5. a**)** Full scans of qPCR gels in main Figure 5a.

Supplementary Figure 3

a

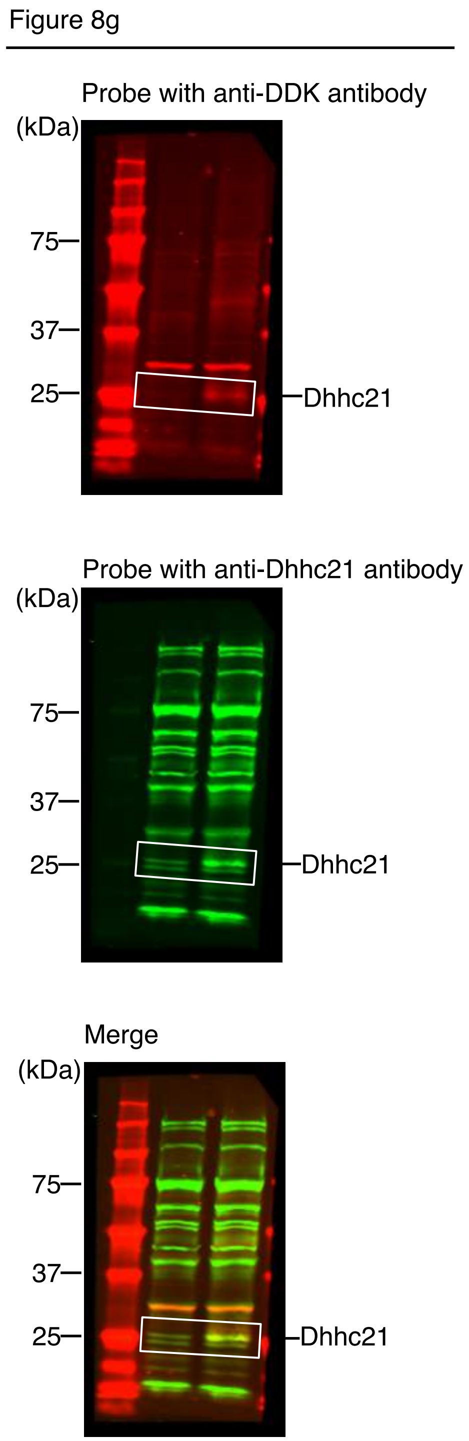

Supplementary Figure 3: Full-scanned images of western blots in main Figure 8. a) Full scans of blots in main Figure 8g.

Supplementary Figure 4

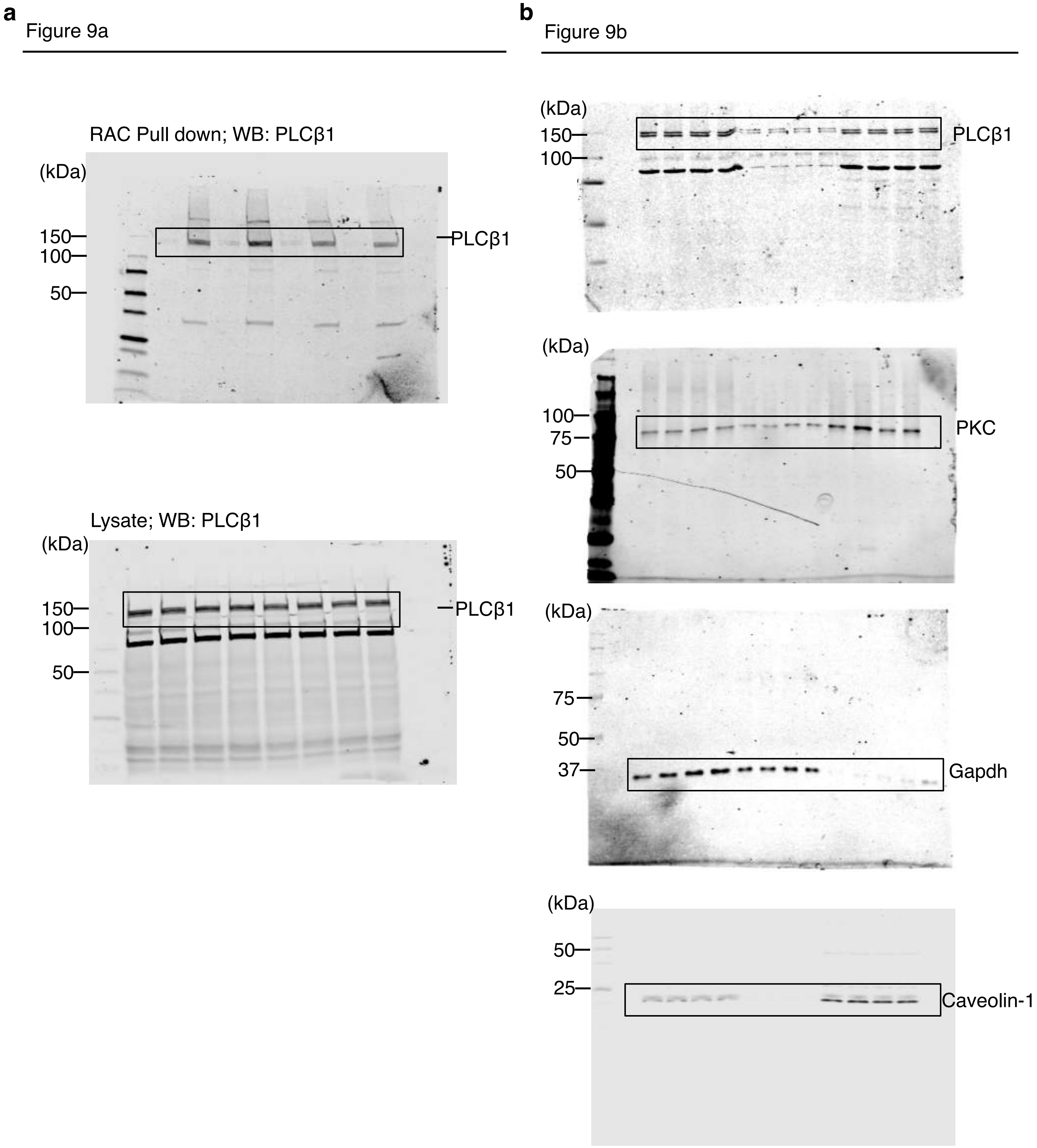

Supplementary Figure 4: Full-scanned images of western blots in main Figure 9. a**)** Full scans of blots in main Figure 9a. b**)** Full-scans of blots in main Figure 9b.

Supplementary Figure 5

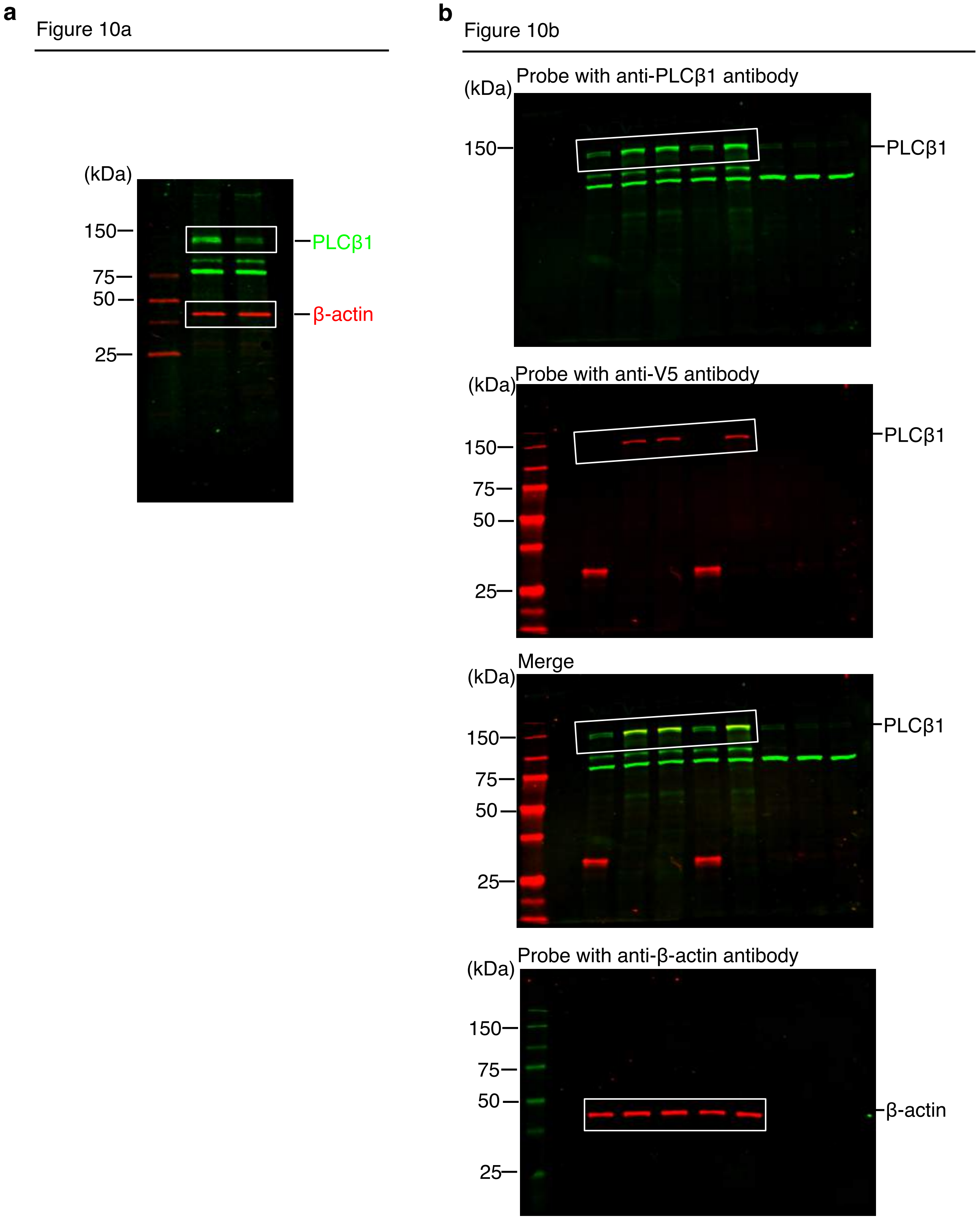

Supplementary Figure 5: Full-scanned images of western blots in main Figure 10. a**)** Full scans of blots in main Figure 10a. b**)** Full scans of blots in main Figure 10b.

SUPPLEMENTARY TABLE 1

Gene	Human Alternatives	Mouse Alternatives
ZDHHC 1	C16orf1, ZNF377, HSU90653	
ZDHHC 2	REAM, REC, ZNF372	
ZDHHC 3	ZNF373, HSD49, GODZ	Godz, Gramp1
ZDHHC 4	ZNF374, DC1, UNQ5787/PRO19576, FLJ10479	
ZDHHC 5	KIAA1748, ZNF375	Kiaa1748
ZDHHC 6	ZNF376, FLJ21952	
ZDHHC 7	ZNF370, FLJ10792, ZNF370, FLJ20279, SERZ-B, SERZ1	Gramp2
ZDHHC 8	KIAA1292, ZDHHCL1, ZNF378	
ZDHHC 9	CXorf11, ZDHHC10, ZNF379, ZNF380, CGI-89, UNQ261/PRO298	
ZDHHC 11	ZNF399, FLJ13153	
ZDHHC 11b		HUMAN ONLY
ZDHHC 12	ZNF400, PSEC0008, FLJ14524	
ZDHHC 13	HIP14L, HIP3RP, FLJ10852, FLJ10941	Hip14l
ZDHHC 14	FLJ20984, NEW1CP	
ZDHHC 15	UNQ1969/PRO4501, FLJ31812, MRX91	
ZDHHC 16	UNQ2570/PRO6258, APH2	Aph2
ZDHHC 17	HIP14, HIP3, HYPH, KIAA0946, HSPC294	Hip14, Kiaa0946
ZDHHC 18	DKFZp667O2416	
ZDHHC 19	MGC3345	Gm616
ZDHHC 20	FLJ25952	
ZDHHC 21	HSPC097, DNZ1	Gramp3
ZDHHC 22	C14orf59	Gm262
ZDHHC 23	MGC42530	Gm779
ZDHHC 24	UNQ2528/PRO6027	
ZDHHC 25	MOUSE ONLY	mCG_52425

SUPPLEMENTARY TABLE 2

Reagent/Drug	Company	Catalogue Number
2-BP (2-bromohexadecanoic acid)	Sigma	238422
15-hexadecynoic acid (Alk-C16)	Avanti	900400
4×protein Loading Buffer	Li-Cor	928-40004
96 well Greiner Black Plate	Greiner Bio-one	675097
Acetone	Fisher	A181
Acridine Orange	Sigma	158550
Activated Charcoal	Sigma	242276
Agarose beads, azide functional	Nanocs	AR-AZ-1
Albumin–fluorescein isothiocyanate conjugate	Sigma	A9771
Alexa Fluor 647 Phalliodin	Life Technologies	A22287
Amaxa P5 Primary Cell 4D-Nucleofector X Kit	Lonza	V4XP-5024
β-mercaptoethanol	MP Biomedicals LLC	194834
BCA Assay	Fisher	PI23227
Biotin-HPDP	Thermo Scientific	21341
CellMask Deep Red Stain	Life Technologies	C10046
Cell Tag 680	Li-Cor	926-41090
Complete mini EDTA free Protease inhibitor cocktail	Roche	4693159001
Copper (II) sulfate pentahydrate	Sigma	209198
Chloroform	Acros Organics	N/A
CellTracker Green	Life Technologies	C7025
Dextran	Sigma	31390
DTT	Fisher	BP172
EBM-2 Basal Endothelial Growth Medium	Lonza	CC-3156
ECIS electrode array	Applied Biophysics	8W10E+
EGM-2 MV BulletKit	Lonza	CC-3202
Endothelial Cell Medium	Cell Biologics	M1168-Kit
Evan's blue dye	Sigma	E2129
FITC-Albumin	Sigma	A9771
Fluo-4 Calcium Imaging Kit	Life Technologies	F10489
Gelatin Solution (0.1%)	ATCC	PCD-999-027
GeneJET Plasmid Miniprep Kit	Thermo Scientific	K0502
Hanks Balanced Salt Solution	Life Technologies	14025-092
Heparin	Vivarium	n/a
Histamine dihydrocholoride	Tocris	3454
Histopaque 1077/1119	Sigma	10771/11191
Hydroxylamine (HA)	Sigma	159417
Hydroxymyristic Acid	Sigma	H6771
Isoflurane	Piramal Healthcare	NDC 66794-017-25
IP3 ELISA Kit	MyBioSource	MBS024296
iScript cDNA Synthesis kit	Bio-rad	170-8891
Kapa2G Fast Hotstart 2X Readymix	Kapa Biosystems	KK5601

**Lactated Ringer's Injection, USP**	**Hospira**	**0409-7953-03**
***E. coli* LPS**	**Sigma**	**L4005**
**Methanol**	**Fisher**	**N/A**
**Methylmethanethiosulfonate (MMTS)**	**Sigma**	**208795**
**Mouse Endothelial Cell Medium Supplement Kit**	**Cell Biologics**	**M1168-Kit**
**Odyssey Protein Molecular Weight Marker**	**Li-Cor**	**928-40000**
**Oregon Green 488 Azide**	**Life Technologies**	**O10180**
**Palmostatin B (ATP1 inihibitor)**	**EMD Millipore**	**178501**
**ProLong Diamond Antifade Mountant with DAPI**	**Life Technologies**	**P36962**
**Protein G Magnetic Beads**	**Thermo Scientific**	**88847**
**Paraformaldehyde**	**Acros**	**41678**
**Plasma Membrane Protein Extraction Kit**	**Abcam**	**ab65400**
**Purelink Hipure Plasmid Filter Maxiprep Kit**	**Life Technologies**	**K210016**
**Purelink Hipure Precipitator Module**	**Life Technologies**	**K2100-21**
**Q5 Site-Directed Mutagenesis Kit**	**New England Biolabs**	**E0554S**
**RIPA**	**EMD Millipore**	**20-188**
**RNA Nano Chips**	**Agilent Tecnologies**	**5067-1511**
**RNAzol**	**Molecular Research Center**	**RN 190**
**SsoAdvanced Universal SYBR Green Supermix**	**Bio-rad**	**172-5272**
**Strepavidin-Magnetic Beads**	**Sigma**	**88816**
**Streptavidin IRdye-800**	**Li-Cor**	**926-32230**
**Streptavidin Texas-Red**	**Vector**	**SA-5006**
**Sulfo–N-hydroxysuccinimide (NHS)–biotin**	**Fisher**	**PI21335**
**Tris[(1-benzyl-1H-1,2,3-triazol-4-yl)methyl] amine (TBTA)**	**Sigma**	**678937**
**TCEP-HCl**	**Thermo Fisher Scientific**	**20490**
**Thrombin**	**Sigma**	**T7513**
**Thiopropyl Sepharose 6B**	**GE Healthcare**	**17-0420-17**
**Transwell 6.5mm with 0.4μM pore PC membrane**	**Corning**	**3413**
**Urethane**	**Sigma**	**94300**
**Vectashield Mounting Medium with DAPI**	**Vecotr Laboratories**	**H-1200**

SUPPLEMENTARY TABLE 3

Antibody	Species	Application	Company	Catalogue No.	Working Concentration
Caveolin-1	Rabbit	Control for Plasma Membrane Fraction	Sigma	C4490	1:500
DDK (FLAG)	Mouse	Overexpression Confirmation	Origene	TA50011-100	1:1000
ICAM-1	Mouse	ICC for HUVECs	Sigma	519369	1:100
ICAM-1	Rat	On-cell Western Assay for MLMVECs	eBioscience	14-0541-85	1:100
GAPDH (14C10)	Rabbit	Control for Cytoplasm Fraction	Cell Signaling	2118	1:500
VE-cadherin (C-19)	Goat	Immunocytochemistry	Santa Cruz	Sc-6458	1:200
Donkey anti-Mouse Alexa Fluor 488	Donkey	Immunocytochemistry	Life Technologies	A21202	1:500
Donkey anti-Goat Alexa Fluor 568	Donkey	Immunocytochemistry	Life Technologies	A11057	1:500
Goat anti-Rat IRDye 800CW	Goat	On-cell Western Assay for MLMVECs	Licor	926-32219	1:200
PKC (H-300)	Rabbit	Plasma Membrane Localization	Santa Cruz	sc-10800	1:500
PLCβ1 (16)	Mouse	Western Blotting	Santa Cruz	sc-136040	1:500
IRDye 800CW Donkey anti-Mouse	Donkey	Western Blotting	Licor	926-32212	1:20000
IRDye 680RD Donkey anti-Rabbit	Donkey	Western Blotting	Licor	926-68073	1:20000
IRDye 800CW Donkey anti-Rabbit	Donkey	Western Blotting	Licor	926-32213	1:20000
IRDye 680LT Donkey anti-Mouse	Donkey	Western Blotting	Licor	926-68022	1:20000
V5-Tag (D3H8Q)	Rabbit	Overexpression Confirmation	Cell Signaling	13202	1:500
ZDHHC21	Rabbit	Western Blotting	Abcam	ab103755	1:500

SUPPLEMENTARY TABLE 4

Unigene	*mRNA Gene*	GeneID	PrimePCR Cat#	Template Cat#
Mm.391967	*Actb*	qMmuCEP0039589	100-25636	100-29101
n/a	*DNA Ctrl*	qMmuCtlD0001004	100-25352	n/a
Mm.304088	*Gapdh*	qMmuCED0027497	100-25636	100-29101
n/a	*PCR pos. Ctrl*	qMmuCtlD0001003	100-25591	n/a
n/a	*RT Ctrl*	qMmuCtlD0001001	100-25695	n/a
Mm.3360	*Ywhaz*	qMmuCED0027504	100-25636	100-29101
Mm.100917	*Zdhhc1*	qMmuCID0022947	100-25636	100-29101
Mm.34326	*Zdhhc2*	qMmuCED0048940	100-25636	100-29101
Mm.28300	*Zdhhc3*	qMmuCED0040569	100-25636	100-29101
Mm.261606	*Zdhhc4*	qMmuCED0045364	100-25636	100-29101
Mm.288508	*Zdhhc5*	qMmuCED0047534	100-25636	100-29101
Mm.386789	*Zdhhc6*	qMmuCID0039678	100-25636	100-29101
Mm.240076	*Zdhhc7*	qMmuCID0012266	100-25636	100-29101
Mm.326263	*Zdhhc8*	qMmuCID0011266	100-25636	100-29101
Mm.207367	*Zdhhc9*	qMmuCID0012400	100-25636	100-29101
Mm.66995	*Zdhhc11*	qMmuCID0021647	100-25636	100-29101
Mm.22538	*Zdhhc12*	qMmuCED0045268	100-25636	100-29101
Mm.279116	*Zdhhc13*	qMmuCID0010336	100-25636	100-29101
Mm.399660	*Zdhhc14*	qMmuCID0027297	100-25636	100-29101
Mm.30574	*Zdhhc15*	qMmuCID0013012	100-25636	100-29101
Mm.20387	*Zdhhc16*	qMmuCID0010210	100-25636	100-29101
Mm.339281	*Zdhhc17*	qMmuCID0040134	100-25636	100-29101
Mm.331948	*Zdhhc18*	qMmuCID0022805	100-25636	100-29101
Mm.76859	*Zdhhc19*	qMmuCID0009286	100-25636	100-29101
Mm.29044	*Zdhhc20*	qMmuCED0046193	100-25636	100-29101
Mm.32047	*Zdhhc21*	qMmuCED0048001	100-25636	100-29101
Mm.185890	*Zdhhc22*	qMmuCID0017666	100-25636	100-29101
Mm.327852	*Zdhhc23*	qMmuCID0017162	100-25636	100-29101
Mm.36885	*Zdhhc24*	qMmuCID0009519	100-25636	100-29101
Mm.45306	*Zdhhc25*	qMmuCED0002084	100-25636	100-29101

SUPPLEMENTARY TABLE 5

siRNA and plasmids	Company	Catalogue #
Control siRNA-A	Santa cruz	sc-37007
Control siRNA-B	Santa cruz	sc-44230
Control siRNA (FITC Conjugate)-A	Santa cruz	sc-36869
pCMV6-Entry (Myc-DDK tag)	Origene	PS100001
pCMV6-Zdhhc21 (Myc-DDK tag)	Origene	MR203515
PLCβ1 siRNA (m)	Santa cruz	sc-36267
pLX304-EGFP (V5 tag)	GeneCopoeia	EX-EGFP-LX304
pLX304-PLCβ1 (V5 tag)	GeneCopoeia	EX-OL07859-LX304
Silencer GAPDH siRNA neg ctl	Life Technologies	AM4624
Silencer negative control no. 1 siRNA	Life Technologies	AM4611
Silencer select siRNA s50719	Life Technologies	4392420
ZDHHC2 siRNA	Santa cruz	sc-155497
ZDHHC4 siRNA	Santa cruz	sc-155504
ZDHHC5 siRNA	Santa cruz	sc-155505
ZDHHC8 siRNA	Santa cruz	sc-155508
ZDHHC9 siRNA	Santa cruz	sc-155509
ZDHHC12 siRNA	Santa cruz	sc-155491
ZDHHC13 siRNA	Santa cruz	sc-75258
ZDHHC16 siRNA	Santa cruz	sc-155494
ZDHHC17 siRNA	Santa cruz	sc-75258
ZDHHC20 siRNA	Santa cruz	sc-155498
ZDHHC21 siRNA	Santa cruz	sc-155499

SUPPLEMENTARY TABLE 6

Figure Panel	Species	Strain	Sex	Replicates (n=)	Age (weeks)
Fig. 1a-b	Rat	Sprague-Dawley	Male	6	9.1±1.2
Fig. 1c-d	Rat	Sprague-Dawley	Male	6	9.5±2
Fig. 1e	Rat	Sprague-Dawley	Male	3	9.5
Fig. 3a-b	Rat	Sprague-Dawley	Male	6	9.5±2
Fig. 6a,c	Mouse	Zdhhc21$^{+/+}$(B6C3Fe); Zdhhc21$^{dep/dep}$	Male	5	12±1
Fig. 6b,e	Mouse	Zdhhc21$^{+/+}$(B6C3Fe); Zdhhc21$^{dep/dep}$	Male	5	12±1
Fig. 6d	Mouse	Zdhhc21$^{+/+}$(B6C3Fe); Zdhhc21$^{dep/dep}$	Mix	15 (Male=8; Female=7)	12±2
Fig. 6f	Mouse	Zdhhc21$^{+/+}$(B6C3Fe); Zdhhc21$^{dep/dep}$	Mix	10 (Male=5; Female=5)	12±2
Fig. 7a-b	Mouse	Zdhhc21$^{+/+}$(B6C3Fe); Zdhhc21$^{dep/dep}$	Male	6	12±1
Fig. 8a-b	Mouse	Zdhhc21$^{+/+}$(B6C3Fe); Zdhhc21$^{dep/dep}$	Male	4	12.5
Fig. 8c-d	Mouse	Zdhhc21$^{+/+}$(B6C3Fe); Zdhhc21$^{dep/dep}$	Mix	6 (Male=3; Female=3)	5.9±1.1

SUPPLEMENTARY TABLE 7

Figure Panel	Statistical Test	Post-hoc Test
Fig. 1b	Two-way ANOVA Repeated Measures	Bonferroni
Fig. 1d	One-way ANOVA	Tukey’s
Fig. 2a	One-way ANOVA	Tukey’s
Fig. 2c,f	One-way ANOVA	Newman-Keuls
Fig. 2d	Two-way ANOVA Repeated Measures	Sidak
Fig. 3b,d,f	One-way ANOVA	Tukey's
Fig. 4a	One-way ANOVA	Newman-Keuls
Fig. 4b,d	One-way ANOVA	Tukey’s
Fig. 4c	T-test (two-sided)	N/A
Fig. 5b	T-test (two-sided)	N/A
Fig. 5d,f	One-way ANOVA	Dunnett's
Fig. 6c,e	One-way ANOVA	Tukey’s
Fig. 6d,f	Log-Rank (Mantel-Cox) test	N/A
Fig. 7b (Burn)	T-test (two-sided)	N/A
Fig. 7b (LPS)	T-test (two-sided)	N/A
Fig. 7d	One-way ANOVA	Tukey’s
Fig. 8b,e,f	One-way ANOVA	Tukey’s
Fig. 8d	Two-way ANOVA Repeated Measures	Bonferroni
Fig. 8g	T-test (two-sided)	N/A
Fig. 9c	One-way ANOVA	Tukey's
Fig. 10a	T-test (two-sided)	N/A
Fig. 10c-f	One-way ANOVA	Tukey’s
